# Unveiling the Genetic Landscape of *Staphylococcus aureus* Isolated From Hospital Wastewaters: Emergence of Hypervirulent CC8 Strains in Tehran, Iran

**DOI:** 10.1155/ijm/5458315

**Published:** 2025-03-13

**Authors:** Fatemeh Sadat Tabatabaie Poya, Mirmohammad Miri, Zahra Salehi, Mohammad Javad Nasiri, Masoud Dadashi, Mehdi Goudarzi

**Affiliations:** ^1^Department of Microbiology, School of Medicine, Shahid Beheshti University of Medical Sciences, Tehran, Iran; ^2^Department of Critical Care and Anesthesiology, Imam Hossein Hospital, Shahid Beheshti University of Medical Sciences, Tehran, Iran; ^3^Department of Mycology, Pasteur Institute of Iran, Tehran, Iran; ^4^Department of Microbiology, School of Medicine, Alborz University of Medical Sciences, Karaj, Iran

**Keywords:** hospitals, methicillin-resistant *Staphylococcus aureus*, *Staphylococcus aureus*, vancomycin-resistant *Staphylococcus aureus*, wastewaters

## Abstract

**Objective(s):** Multidrug-resistant bacteria and priority pathogens, including MRSA, are frequently found in hospital wastewaters. It is crucial to investigate the genetic diversity, biofilm formation, and virulence analysis of *Staphylococcus aureus* isolated from hospital wastewaters.

**Materials and Methods:** In this cross-sectional study, 70 *S. aureus* isolated from hospital wastewaters were subjected to characterization through antimicrobial susceptibility tests, biofilm formation, multilocus sequence typing (MLST), and PCR analysis for detecting resistance (*mecA*, *mecC*, *vanA*, *vanB*, *mupB*, *mupA*, *msr(A)*, *msr(B)*, *erm*(A), *erm*(B), *erm*(C), *tet*(M), *ant* (4⁣′)*-Ia*, *aac* (6⁣′)*-Ie/aph* (2⁣^″^), and *aph* (3⁣′)*-IIIa*) and virulence genes (*eta*, *etb*, *pvl*, and *tst*).

**Results:** Our results showed that 55.7%, 31.4%, and 12.9% of isolates were classified as strong, intermediate, and weak biofilm-forming strains, respectively. Our result revealed that about three-quarters of isolates harbored *mecA* (100%), *ant* (4⁣′)*-Ia* (100%), *tet*(M) (92.9%), *erm*(B) (80%), and *msr*(A) (74.3%) resistance genes. MLST revealed that the 70 isolates belonged to five clonal complexes, including CC8 (52.9%), followed by CC30 (15.7%), CC5 (14.3%), CC1 (11.4%), and CC22 (5.7%). The vast majority of *S. aureus* isolates belonged to CC8/ST239-MRSA (21.5%). Among the 39 strong biofilm producers, the majority (25.6%) belonged to CC8/ST239-MRSA clone. Our result revealed that about one-third of Panton–Valentine leukocidin (PVL)–positive strains belonged to CC30/ST30. The high-level mupirocin-resistant (HLMUPR) isolates belonged to CC8/ST239-MRSA (36%), CC30/ST30-MRSA (16%), CC8/ST8-MRSA (12%), CC5/ST5-MRSA (12%), CC8/ST585-MRSA (8%), CC5/ST225-MRSA (8%), CC5/ST1637-MRSA (4%), and CC8/ST1465-MRSA (4%) lineages carrying *mupA*. The VRSA strain belonged to the CC8/ST239-MRSA, CC8/ST8-MRSA, and CC22/ST22-MRSA clonal lineages, carrying the *vanA* determinant.

**Conclusion:** These findings highlight significant genotypic diversity and high biofilm formation among our isolates. From this study, we identified highly virulent strains of *S. aureus* associated with biofilm production and drug resistance; some of these strains were highly similar, highlighting the possibility of rapid spread. The high prevalence of CC8 and CC30 clones among *S. aureus* strains reflects the emergence of these lineages as successful clones in hospital wastewaters in Iran, which is a serious concern. The study highlights the importance of wastewater surveillance to understand genetic pattern and antimicrobial resistance profiles in surrounding communities, which can in turn support public health efforts.

## 1. Introduction

The prevalence of antibiotic-resistant bacteria is not only common in areas where antibiotics are utilized, but it is also increasingly found in aquatic environments [1]. Literature has indicated that around 30% of hospitalized patients receive antibiotic treatment, resulting in the dissemination of large quantities of antimicrobial drugs and drug-resistant bacteria into the environment. The discharge of active antibiotics into wastewater is significant, making wastewater a crucial reservoir for the development and spread of antibiotic resistance [2]. Healthcare settings continuously generate wastewater that requires proper treatment. Hospital sewage contains a variety of multidrug-resistant (MDR) bacteria, as well as substances like antimicrobials, disinfectants, heavy metals, and nonmetabolized drugs, all of which contribute to the environmental reservoir and play a key role in the spreading of genes that cause antibiotic resistance [1, 2]. The emergence of MDR bacteria into the environment has several implications for public health. The release of resistant bacteria into the environment has several significant implications for public health. Firstly, if these bacteria carry transmissible genes, they can spread these genes to other bacteria in the community. As a result, infections caused by these bacteria become more challenging to treat, and the availability of antibiotics for treating bacterial infections decreases. Secondly, these resistant bacteria may serve as vectors or reservoirs for spreading resistant genes further. Thirdly, there is an increased risk of nosocomial infections (infections acquired in healthcare settings). Lastly, if an infection occurs, the costs associated with treatment and hospitalization will rise [2, 3]. Hospital waste can pose a hazard because it contains infectious agents and toxic substances. Antibiotics are a specific type of substance that is excreted in an unmetabolized form, and when they end up in wastewater, they can contribute to the development of bacteria that are resistant to multiple drugs [1, 3]. This is mainly because hospitals often use antibiotics that are considered a last resort. The concentrations of antibiotics in hospital wastewater are significantly lower than the dosages used for treatment. However, even at these lower concentrations, antibiotics can exert a selective pressure on bacteria, leading to the development of resistance [2, 3]. In addition, the presence of antibiotics in wastewater can facilitate the transfer of resistance genes between bacteria of the same species and different species [1–3]. Several microorganisms are known to be recovered from hospital wastewater. Given the recently reported evidence, *Staphylococcus aureus* is known as the commonest causative agent. In recent years, the frequency of MDR *S. aureus* strains isolated from hospital wastewater has increased, posing a significant challenge in healthcare settings worldwide [2]. Consequently, numerous studies have focused on understanding the molecular characteristics, antibiotic resistance, and biofilm formation patterns of *S. aureus* strains isolated from hospital wastewater in Iran [4, 5]. However, there is still limited knowledge regarding the genetic diversity and biofilm-forming ability of this microorganism. Therefore, our study is aimed at investigating the molecular characteristics, antibiotic resistance, and biofilm formation patterns of *S. aureus* isolates in hospital wastewater.

## 2. Material and Methods

### 2.1. Sample Collection

The wastewater samples were collected weekly over 15 months between June 2022 and August 2023. A total of 70 samples were obtained from the wastewater of two teaching hospitals affiliated with Shahid Beheshti University of Medical Sciences. The samples were collected weekly, with three samples collected each day in sterile 500-mL plastic bottles containing sodium thiosulfate, and then stored at 4°C. About 100 mL of hospital wastewater was filtered through a membrane with a pore size of 450 nm and then cultured on blood agar (HiMedia, Mumbai, India).

### 2.2. *S. aureus* Isolation and Identification

The golden or white colonies were identified through the evaluation of catalase (HiMedia, Mumbai, India) and coagulase (HiMedia, Mumbai, India) production using the tube method with rabbit-citrated plasma. For further analysis, the colonies were then cultivated on mannitol-salt agar (HiMedia, Mumbai, India) and DNase mediums (HiMedia, Mumbai, India). The genetic confirmation of isolates was achieved through the detection of the *nucA* gene (270 bp) using polymerase chain reaction (PCR) with forward and reverse primer sequences of F: GCGATTGATGGTGATACGGTT and R: AGCCAAGCCTTGACGAACTAAAGC. The PCR products were electrophoresed on 1.5% agarose gels. Confirmed *S. aureus* isolates were cultured in tryptic soy broth (TSB) (HiMedia, Mumbai, India) containing 20% sterile glycerol and were stored at −70°C [6].

### 2.3. Determination of Isolate Susceptibility

The susceptibility of the isolates was evaluated through the Kirby–Bauer method, adhering to the Clinical and Laboratory Standards Institute (CLSI) criteria. This method was employed to assess the resistance profile of the isolates against antibiotics, such as clindamycin, nitrofurantoin, rifampin, erythromycin, ciprofloxacin, penicillin, gentamicin, and tetracycline. These antibiotics are indeed primary lines of defense against *S. aureus* infections. The minimum inhibitory concentration (MIC) values for vancomycin and mupirocin (both low-level and high-level resistances) were determined using the microtiter broth microdilution method as previously reported. The MIC and *D*-zone tests were conducted in accordance with the CLSI criteria. Methicillin resistance was assessed using a cefoxitin disc (30 *μ*g) on Mueller–Hinton agar plates in accordance with CLSI guidelines (CLSI 2022). The experiment was controlled by utilizing reference strains of *S. aureus*American Type Culture Collection (ATCC) 29213, ATCC 43300, and ATCC 25923.

### 2.4. Microtiter Plate (MtP) Assay

The biofilm formation assay was performed as previously described (8). In this method, a 1:10 dilution of an overnight culture of *S. aureus* in TSB (HiMedia, Mumbai, India) supplemented with 1% glucose (Merck, Germany) was prepared at 37°C. Individual wells were filled with 200 *μ*L of the dilution before incubation for 24 h at 37°C under static conditions without shaking. Following incubation, the suspended bacteria were removed by gentle tapping and washing three times with 200 *μ*L phosphate buffer saline (PBS; pH 7.2). Adherent bacteria were fixed with 99% methanol for 15 min, air-dried for 20 min, and stained with 200 *μ*L safranin solution (0.1%) for 5 min. Then, the wells were rinsed four times with distilled water to remove excess stains. The adhered bacterial cells were resolubilized in 200 *μ*L of 33% (*v*/*v*) glacial acetic acid (Merck, Germany) and incubated at 37°C for 15 min. TSB supplemented with 1% glucose was used as the negative control. The optical density (OD) of the stained biofilm was measured at a 490-nm wavelength using an ELISA. Calculation of the mean OD + 3 × standard deviation (SD) of blank control samples yielded an OD cut-off (ODc) value, used in further classifying the strains into four categories based on biofilm production: no biofilm (OD ≤ 0.059), weak (ODc < OD ≤ 2 × ODc), moderate (2 × ODc < OD ≤ 4 × ODc), and strong (OD > 4 × ODc) [7]. The *Staphylococcus epidermidis* ATCC 35984 strain was used as a positive control for biofilm formation. Control positive sample for biofilm formation included *S. epidermidis* ATCC 35984 strain.

### 2.5. Extraction of Genomic DNA and Detection of Toxin and Resistance Encoding Determinants

The genomic DNA was obtained using phenol–chloroform technique, as explained earlier [8]. The presence of the toxin (*eta*, *etb*, *pvl*, and *tst*) and resistance (*erm*(B), *tet*(M), *mecC*, *msr(B)*, *mecA*, *mupA*, *vanA*, *aac* (6⁣′)*-Ie/aph* (2⁣^″^), *mupB*, *msr(A)*, *erm*(C), *erm*(A), *vanB*, *ant* (4⁣′)*-Ia*, and *aph* (3⁣′)*-IIIa*) encoding genes was identified by the PCR method using fragment-specific primers [6]. The primer sequences were also checked by Basic Local Alignment Search Tool (BLAST) service available on the National Center for Biotechnology Information (NCBI) GenBank website (https://blast.ncbi.nlm.nih.gov/Blast.cgi). The PCR assay was carried out in a final volume of 25 *μ*L, containing 18 *μ*L of Taq DNA polymerase master mix (SinaClon, Tehran, Iran), 5 *μ*L of DNA template (50 ng), and 1 *μ*L of each forward and reverse primers (10 pM). PCR products were analyzed using electrophoresis on 1% agarose gel. Also, DNA bands were visualized by staining via ethidium bromide and photographed under UV illumination. The GeneRuler 100 bp Plus DNA Ladder (Fermentas, Vilnius, Lithuania) was used as a molecular size marker.

### 2.6. Multilocus Sequence Typing (MLST)

All isolates underwent the MLST assay following the established protocol, described by Rahmani et al. [9]. This assay included the utilization of primers, PCR reaction conditions, and specific procedures. The identification of allele profiles and sequence types (STs) was achieved by comparing the acquired sequences of housekeeping genes (*tpiA*, *arcC*, *gmK*, *glpF*, *aroE*, *yqiL*, and *pta*) with the information available on the MLST database website (https://pubmlst.org/). The eBURST program version 3 was utilized to determine the clustering of related STs, referred to as a clonal complex (CC). CC for *S. aureus* is also provided by searching for the ST on PubMLST. Negative DNA control (PCR-grade water) was included in all sets of PCR. The PCR products were electrophoresed in a 0.5% agarose gel containing Tris/borate/EDTA (TBE) running buffer at 80 V for 60 min, then photographed under UV light by a gel documentation system (UVItec, United Kingdom). The GeneRuler 100 bp Plus DNA Ladder (Fermentas, Vilnius, Lithuania) was used as a molecular size marker.

## 3. Results

### 3.1. Identification and Antibacterial Susceptibility Pattern

**Figure 1:**
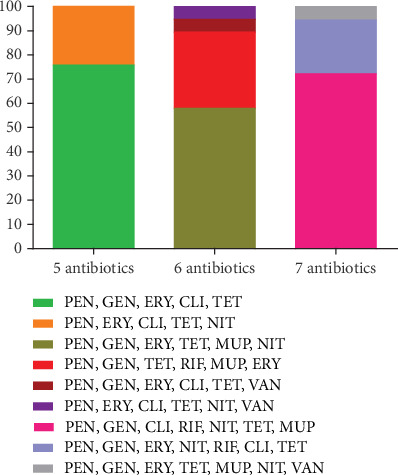
Antibiotic resistance patterns of 70 *S. aureus* isolated from hospital wastewater. ERY, erythromycin; VAN, vancomycin; MUP, mupirocin; GEN, gentamicin; NIT, nitrofurantoin; TET, tetracycline; CLI, clindamycin; PEN, penicillin; RIF, rifampicin.

In present survey, 70 isolates of *S. aureus* obtained from hospital wastewater samples were used. Overall, susceptibility to all of the antibacterial agents was not detected among the examined strains. All strains were resistant to penicillin. The highest level of antibiotic resistance was observed against tetracycline (94.3%), followed by gentamycin (87.1%), erythromycin (81.4%), clindamycin (74.3%), nitrofurantoin (54.3%), mupirocin (44.3%), rifampicin (32.9%), and vancomycin (4.3%). In total, all of the isolates were found to be methicillin-resistant *S. aureus* (MRSA) and MDR. Extensively drug-resistant (XDR) *S. aureus* was identified in our study. We found nine resistance patterns in the examined isolates (Figure 1). In our study, 14 resistance patterns were detected, wherein penicillin (PEN), gentamicin (GEN), erythromycin (ERY), clindamycin (CLI), tetracycline (TET) (35.7%; 25/70); PEN, GEN, CLI, rifampin (RIF), nitrofurantoin (NIT), TET, mupirocin (MUP) (18.6%; 13/70); and PEN, GEN, ERY, TET, MUP, NIT (15.7%; 11/70) were the top three frequently detected profile. In the current research, 18 (25.7%) of the isolates were confirmed to have inducible clindamycin resistance (ICR). All the ICR strains were resistant to mupirocin. Constitutive clindamycin-resistant (CCR) isolates were detected at a prevalence rate of 55.7%. According to our data, all the CCR isolates were found to be resistant to tetracycline. The present analysis revealed that 25 isolates (35.7%) had high-level mupirocin resistance (HLMUPR) and six isolates (8.6%) had low-level mupirocin resistance (LLMUPR) pattern. Out of the 70 *S. aureus* isolates which were tested, three *S. aureus* isolates were resistant to vancomycin (4.3%). In present research, 24 isolates (34.3%) were inhibited by 1 *μ*g/mL of vancomycin, 43 isolates (61.4%) by 2 *μ*g/mL, and 3 isolates (4.3%) by 32 *μ*g/mL.

Of the three vancomycin-resistant *S. aureus* (VRSA) isolates, two isolates and one isolate indicated CCR and ICR phenotype respectively. The data of the microbroth dilution method illustrated that 31 isolates (44.3%) were mupirocin resistant; of these, 25 (80.6%; 25/31) and 6 (19.4%; 6/31) isolates were HLMUPR and LLMUPR, respectively. Of the 31 mupirocin-resistant *S. aureus*, one had MIC values of 64 *μ*g/mL (3.2%), five had a MIC of 128 *μ*g/mL (16.1%), 15 had a MIC of 256 *μ*g/mL (48.4%), eight (25.8%) had a MIC ≥ 512*  μ*g/mL, and two (6.5%) had MIC ≥ 1024*  μ*g/mL.

### 3.2. Biofilm Formation

**Figure 2:**
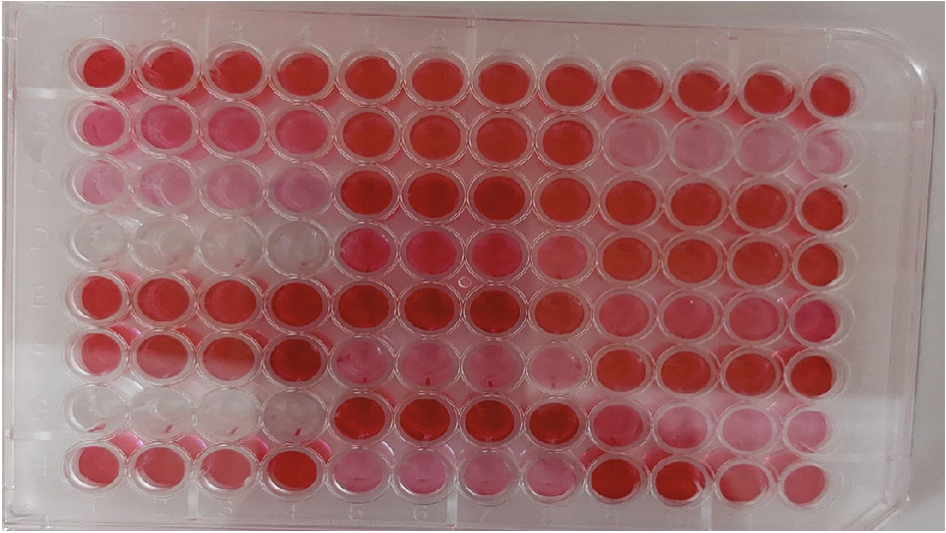
Results of microtiter plate (MTP) assay. Column A 1–4 represent positive control (*S. epidermidis* ATCC 35984 strain), B 1–4 represent moderate biofilm formation, C 1–4 represent weak biofilm formation, D 1–4 represent negative control (sterile tryptic soy broth medium), and E 1–4 represent the strong biofilm formation.

By the MtP assay, all of isolates were found to be able to produce biofilms to different degrees (Figure 2). Out of the isolates that were positive for biofilm formation, 39 (55.7%) were categorized as strong, 22 (31.4%) as moderate, and 9 (12.9%) as weak in their biofilm-forming ability. As presented in Table 1, out of the three VRSA isolates, two showed a high ability for forming biofilm, while one had a moderate ability to form biofilm. All the isolates that exhibited the HLMUPR phenotype were classified as strong biofilm producers. Out of 22 moderate and nine weak biofilm producer isolates, two isolates and one isolate indicated resistance to mupirocin at low level respectively.

### 3.3. Resistance Gene Detection

**Figure 3:**
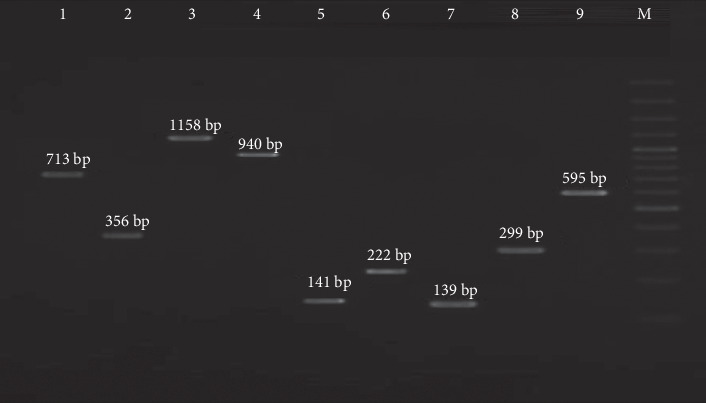
Lane M, 100-bp DNA ladder (Fermentas, London, United Kingdom); Lane 1 *vanA*; Lane 2 *mecC*; Lane 3 *mupA*; Lane 4 *msrA*; Lane 5 *ermB*; Lane 6 *aac (6*⁣′*)-Ie/aph(2*⁣^″^); Lane 7 *ermA*; Lane 8 *ermC*; Lane 9 *msrB*.

According to our results, three MRSA strains were resistant to vancomycin with a MIC ≥ 32*  μ*g/mL. The *vanA* gene was identified in all three VRSA isolates. All VRSA isolates were resistant to PEN, ERY, NIT, and TET. Our results showed that all HLMUPR isolates harbored the *mupA* gene. In this investigation, the findings indicated that all erythromycin and gentamycin-resistant MRSA strains carried at least one macrolide and aminoglycoside resistance gene. Resistance to macrolides was mainly due to *erm*(B), which was present in 56 isolates (80%), followed by *msr*(A) in 52 isolates (74.3%), *erm*(A) in 29 isolates (41.4%), *msr*(B) in 26 isolates (37.1%), and *erm*(C) in 21 isolates (30%). Resistance to aminoglycoside was mainly due to *ant (4*⁣′*)-Ia*, (100%; 70/70), followed by *aac (6*⁣′*)-Ie/aph (2*⁣^″^) (27.1%; 19/70), and *aph (3*⁣′*)-IIIa* (14.3%; 10/70). Our data confirmed the presence of *erm*(A), *erm*(B), *erm*(C), *msr*(A), and *msr*(B) in 94.4%, 33.3%, 55.6%, 55.6%, and 44.4% of ICR isolates. The obtained data also showed the presence of *erm*(A), *erm*(B), *erm*(C), *msr*(A), and *msr*(B) in 20.5%, 84.6%, 7.7%, 74.4%, and 17.9% of CCR isolates. The resistance encoding genes are shown in Figure 3.

### 3.4. MLST

In the current research, all *S. aureus* strains were successfully typed using the MLST technique. A total of 13 particular STs, including ST239 (21.6%; 15/70), ST30 (12.9%; 9/70), ST8 (11.5%; 8/70), ST5 (8.6%; 6/70), ST1465 (7.1%; 5/70), ST585 (7.1%; 5/70), ST1 (7.1%; 5/70), ST421 (5.7%; 4/70), ST22 (5.7%; 4/70), ST225 (4.3%; 3/70), ST772 (4.3%; 3/70), ST36 (2.8%; 2/70), and ST1637 (1.4%; 1/70), were identified and categorized into five CCs. Table S1 gives a summary data of nucleotide sequence among alleles of seven MLST housekeeping genes of *S. aureus* isolates. Based on our results, 13 lineages including CC8/ST239-MRSA (21.5%), CC30/ST30-MRSA (12.9%), CC8/ST8-MRSA (11.5%), CC5/ST5-MRSA (8.6%), CC8/ST1465-MRSA (7.1%), CC8/ST585-MRSA (7.1%), CC1/ST1-MRSA (7.1%), CC8/ST421-MRSA (5.7%), CC22/ST22-MRSA (5.7%), CC1/ST772-MRSA (4.3%), CC5/ST225-MRSA (4.3%), CC30/ST36-MRSA (2.8%), and CC5/ST1637-MRSA (1.4%) were identified. Allelic profile of different ST types of *S. aureus* isolates is presented in Table 2. According to our analysis, the most prevalent CC was CC8 (52.9%; 37/70), followed by CC30 (15.7; 11/70), CC5 (14.3%; 10/70), CC1 (11.4%; 8/70), and CC22 (5.7%; 4/70). CC1, CC5, CC8, and CC30 represented different STs. Mupirocin-resistant isolates were primarily found in CC8/ST239-MRSA (29%; 9/31), CC8/ST8-MRSA (19.3%; 6/31), CC30/ST30-MRSA (12.9%; 4/31), CC5/ST5-MRSA (9.7%; 3/31), CC8/ST421-MRSA (9.7%; 3/31), CC2/ST225-MRSA (6.5%; 2/31), CC8/ST585-MRSA (6.5%; 2/31), CC8/ST1465-MRSA (3.2%; 1/31), and CC5/ST1637-MRSA (3.2%; 1/31) clones. In the present study, CC5 corresponded to CC5/ST5-MRSA (12%; 3/25), CC5/ST1637-MRSA (4%; 1/25), and ST225-MRSA (8%; 2/25); CC8 corresponded ST239-MRSA (36%; 9/25), ST8-MRSAIV (12%; 3/25), ST585-MRSAIII (8%; 2/25), and ST1465-MRSAIII (4%; 1/25); and CC/ST30-MRSAIV (16%; 4/25) exhibited HLMUPR phenotype in *S. aureus* carrying *mupA*. The VRSA strains belonged to CC8/ST239-MRSA, CC/ST8-MRSA, and CC/ST22-MRSA clonal lineages and carried the *vanA* determinant. Our results show that ICR *S. aureus* strains were distributed across various lineages, including CC5/ST5-MRSA (16.7%; 3/18), CC5/ST225-MRSA (11.1%; 2/18), CC5/ST1637-MRSA (5.5%; 1/18), CC8/ST8-MRSA (16.7%; 3/18), CC8/ST1465-MRSA (11.1%; 2/18), CC8/ST421-MRSA (16.7%; 3/18), and CC30/ST30-MRSA (22.2%; 4/18).

**Figure 4:**
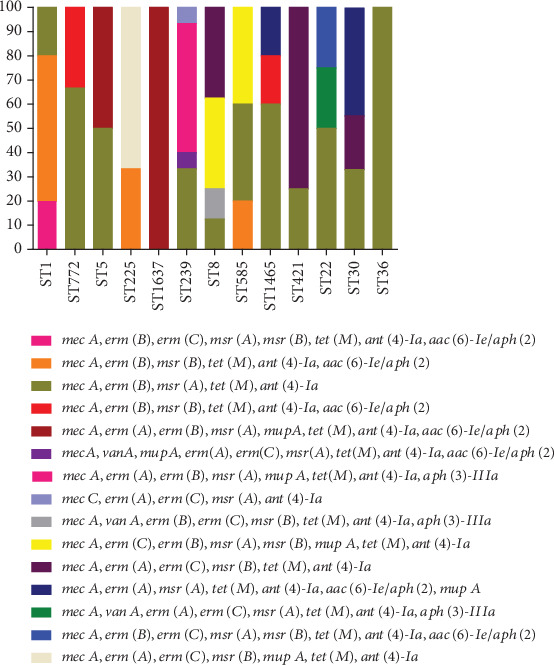
Distribution of antibiotic resistance genes in molecular types of *S. aureus* isolates.

Overall, distribution of CCR *S. aureus* isolates was high in CC8 clonal lineage (22.9%; 16/70). As shown in Figure 4, *mecC* gene was present in one isolate (ST239) (1.4%). *vanA*-positive isolates belonged to ST239, ST8, and ST22 (each one isolate). Moreover, the prevalence of *tst*-positive isolates was limited to 2 CCs including CC5 (6 isolates) and CC8 (12 isolates) while *pvl*-positive MRSA strains belonged to 4 CCs (CC1, 4 isolates; CC8, 6 isolates; CC22, 4 isolates; and CC30, 7 isolates). Our analysis indicated that among the 70 *S. aureus* strains, 2 isolates (2.9%) carried the *eta* and 2 isolates (2.9%) carried *etb* genes. Isolates harboring *eta* gene belonged to ST1465 (1 isolate) and ST36 (1 isolate) while isolates harboring the *etb* gene belonged to ST1637 and ST421 strains (each 1 isolate).

### 3.5. Phylogenetic Analysis

**Figure 5:**
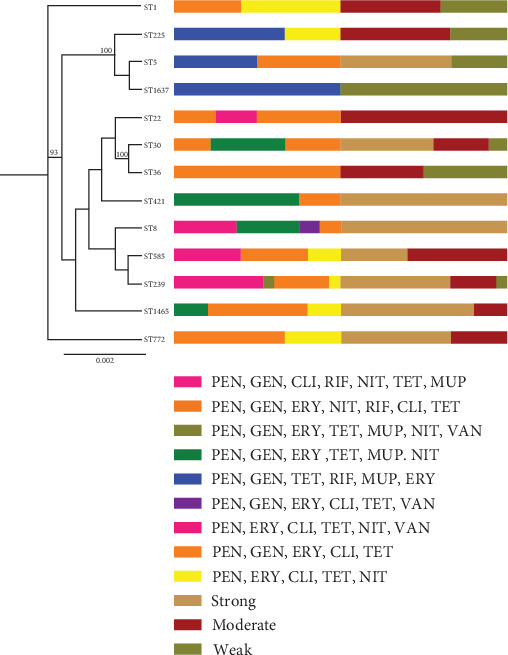
The maximum likelihood phylogenetic tree of combined seven loci (*arcC*, *aroE*, *glpF*, *gmk*, *pta*, *tpi*, and *yqiL*) sequences was constructed using RaxML through the CIPRES science gateway. Values at the nodes represent bootstrap percentages based on 1000 replicates, and branches with bootstrap values exceeding 90% are displayed.

A phylogenetic tree depicting the combined sequences of seven loci (*arcC*, *aroE*, *glpF*, *gmk*, *pta*, *tpi*, and *yqiL*) is presented in Figure 5. The analysis of this phylogenetic tree was performed using RAxML for all STs in this study, utilizing a dataset comprising 3186 bp. The tree effectively illustrates the relationships among STs, antibiotic resistance pattern, and biofilm formation. Notably, ST1 forms a distinct cluster at the top, with ST772 occupying the basal position. Furthermore, the phylogenetic tree distinctly separates ST1 and ST772 from other STs. Bootstrap percentages at the nodes are based on 1000 replicates, and branches with bootstrap values exceeding 90% are highlighted.

## 4. Discussion

In current research, we have identified five CCs including CC8 (52.9%), followed by CC30 (15.7%), CC5 (14.3%), CC1 (11.4%), and CC22 (5.7%). The majority of CCs detected in the present survey have also been declared in earlier various reports as common CCs in *S. aureus* recovered from wastewater [10–12]. A Spanish study on 16 wastewater samples revealed that ST398, ST5, ST126, and ST2849 were the most prevalent clones. They indicated that all ST398 strains were resistant to methicillin [12]. Our findings are similar to those of Silva et al. who reported that the vast majority of *S. aureus* isolates from wastewater belonged to ST22-MRSAIV, ST8-MRSAIV, and ST105-MRSAII clones [11].

The emergence of VRSA poses a significant challenge in controlling staphylococcal infections [13]. It is noteworthy that in the current study, three isolates were found to have reduced susceptibility to vancomycin (4.3%), carrying the *vanA* gene. A meta-analysis conducted by Shariati et al. in 2020 revealed a rising trend of VRSA worldwide. They demonstrated a twofold increase in VRSA cases after 2010 compared to before. Notably, Asian countries had the highest incidence rates of VRSA (67%), potentially due to the easy availability of antibiotics, limited alternative options to vancomycin, inadequate monitoring of drug resistance patterns, and varying attitudes towards antimicrobial protocols [14]. However, the increasing resistance of *S. aureus* to vancomycin raises concerns about the potential emergence of VRSA strains in clinical settings. According to the published data, multidrug resistance (MDR) especially resistance to vancomycin and mupirocin among USA300 strains is increasing globally [15, 16]. Our observations indicated that VRSA isolates belonged to CC8/ST239-MRSA, CC/ST8-MRSA, and CC/ST22-MRSA clonal lineages. CC/ST8-VRSA was earlier reported by Havaei et al. in 2012 [17]. Our analysis also supports the data published from Iran by Akya et al. that reported a low rate of occurrence of VRSA strains carrying *vanA* among *S. aureus* strains recovered from wastewater samples [18]. Earlier, vancomycin-intermediate *S. aureus* (VISA) strains of ST239 have been documented in Pennsylvania [19] and New Zealand [20]. In Iran, Azimian et al. [21] conducted a study who reported that VRSA isolate belonged to ST1283-SCC*mec* III/t037, which is a single-locus variant of ST239, an endemic clone found in many Asian countries. The outcome of this study is in line with earlier research carried out by Goudarzi et al. in Iran that reported CC/ST22-MRSA IV-t790 strain as a common VRSA genotype [22].

Our findings align closely with global data, particularly those from Southeast Asia and Europe [23–25]. In Southeast Asia, *S. aureus* isolates from hospital wastewater are predominantly associated with CC8 and CC30, which were also prominent in our study. Atshan et al. [23] reported that CC8 strains in Malaysia demonstrate robust biofilm-forming capabilities, mirroring the strong biofilm producers observed in our results. Similarly, in Europe, CC22 and CC8 strains are predominant among MRSA populations in both clinical and environmental settings, as documented in studies from Spain and the United Kingdom [24, 25]. European studies, such as those by Silva et al., have similarly documented VRSA emergence in wastewater systems, often linked to endemic clones like ST239 and ST22, which were also detected in this research [12]. These findings highlight the potential risk posed by these isolates in both community and hospital settings, warranting increased attention. Further research with a larger sample size and encompassing various regions of the country is necessary to arrive at a comprehensive conclusion.

Mupirocin can be used to control the spread of *S. aureus* isolates and prevent severe infections in communities and healthcare settings, as reported in earlier researches [26, 27]. Our survey data revealed that 44.3% of examined strains indicated resistance to mupirocin, in which 35.7% and 8.6% indicated HLMUPR and LLMUPR pattern respectively. The prevalence of HLMUPR-MRSA in our work was higher compared to rates reported in Canada (4.3%), China (7%), and France (0.8%) [27, 28]. In the present study, CC5, corresponded to ST5-MRSAIV (12%), ST1637-MRSAIV (4%), and ST225-MRSAII (8%); CC8 corresponded ST239-MRSAIII (36%), ST8-MRSAIV (12%), ST585-MRSAIII (8%), and ST1465-MRSAIII (4%); and CC/ST30-MRSAIV (16%) exhibited HLMUPR phenotype in *S. aureus* carrying *mupA*. These results coincide with those produced in India by Abimanyu, Murugesan, and Krishnan, who reported ST239 as the most predominant type of HLMUPR *S. aureus* causing nosocomial infections [29]. Additionally, in a study conducted by McDougal et al., in the United States, it was reported that the USA300 isolates (CC/ST8) were mupirocin resistant and harbored *mupA* [30]. In similar surveys conducted by Rahmani et al. in Iran, CC/ST15-MRSAIV/t084 (40%), CC/ST22-MRSAIV/t790 (23.3%), CC8/ST239-MRSAIII/t631 (20%), and CC8/ST239-MRSAIII/t030 (16.7%) were most frequently mupirocin-resistant MRSA clones [9]. The examination of resistance genes revealed that out of all the isolates, only one belonged to CC8/ST239-MRSA III and carried the *mecC* gene (1.4%). Similar results have been reported in other regions of the world including Finland, Austria, Switzerland, Spain, Belgium, and United Kingdom [31]. In the United Kingdom and Denmark, the predominance of *mecC* has been observed in CC/ST130 clone [24]. It is noteworthy that prior research of Iran showed that *mecC*-positive isolates belonged to CC/ST130 and CC/ST599 clones [25].

Our findings also displayed a relatively low frequency of ICR *S. aureus* strains (25.7%) which belonged to CC5 (33.3%), CC8 (44.4%), and CC30 (22.2%) lineages. According to our results, resistance to clindamycin was possibly conferred by the *erm*(B) (80%), followed by *msr*(A) (74.3%), *erm*(A) (41.4%), *msr*(B) (37.1%), and *erm*(C) (30%). Several researches have also noted a significant prevalence rate of resistance to macrolides and lincosamides. Current findings in relation to the detection of *erm*(C) gene are in line with the study conducted by Fasihi et al., which reported a 20.5% prevalence of the *erm*(C) gene and 11% of the *erm*(A) gene [32]. The research conducted by Lim et al. demonstrated that out of the different *erm* genes responsible for erythromycin resistance, only *erm*(C) was found to be transferable in transformation experiments. The high prevalence of *msr*(A) gene (74.3%) in our research is in line with research study conducted by Sedaghat et al. (43.6%) [5]. Our observations about *erm*(B) gene indicated a high prevalence rate (80%). The opposite of this occurrence is seen in a study from Texas (46.3%) [33].

In our survey, the data showed that resistance to aminoglycoside was mainly due to *ant (4*⁣′*)-Ia*, (100%), followed by *aac (6*⁣′*)-Ie/aph (2*⁣^″^) (27.1%), and *aph (3*⁣′*)-IIIa* (14.3%). Similar prevalence of *ant (4*⁣′*)-Ia* was reported by Ida et al. from Japan (84.5%) [34] which could be in part due to the horizontal transfer of resistance genes among the MRSA strains. Here, we found a higher level of *ant (4*⁣′*)-Ia* gene compared to what Ardic et al. (24%) in Turkey and Perumal, Murugesan, and Krishnan (9%) in India found before [35, 36]. In the present experiment, the prevalence of *aac (6*⁣′*)-Ie/aph (2*⁣^″^) gene was reported at 27.1%. In a research conducted by Ardic et al., high prevalence rates of *aac (6*⁣′*)-Ie/aph (2*⁣^″^) in *S. aureus* isolates were reported (60.5%%) [35].

According to our data, the *aph (3*⁣′*)-IIIa* gene was detected in 14.3% of isolates. Similarly, Ardic et al. from Turkey (8%) and Akpaka, Roberts, and Monecke from Trinidad and Tobago (9%) previously demonstrated a low prevalence rate of *aph (3*⁣′*)-IIIa* among *S. aureus* isolates [35, 37]. Our findings align with recent reports from the same hospitals, suggesting that the use of aminoglycosides for treating MRSA infections may no longer be effective.

Our findings indicate that a significant proportion of CC8 strains (40%) and a lower percentage of CC30 (7.1%), CC5 (5.7%), and CC1 (2.9%) exhibit a strong ability to form biofilms. These findings are consistent with a study that was carried out in the Netherlands by Croes et al., which also reported a high occurrence of CC8, CC22, CC1, CC30, CC5, and CC45 among strains with strong biofilm-forming capabilities. Specifically, they highlighted the notable biofilm-forming capacity of CC8 strains [38]. Another study conducted by Atshan et al. in Malaysia found that over half of CC8 isolates, and less than one-fifth of CC1, CC22, and CC7 isolates, were identified as frequent biofilm producers [23]. These variations in biofilm production potential among different clonal lineages may be attributed to distinct combinations of surface-associated and regulatory genes [38]. Therefore, based on our results and those of other studies, it is crucial to focus on and effectively manage *S. aureus* isolates with high biofilm-forming abilities.

Although this study provides a detailed analysis of resistance patterns and biofilm-forming capabilities, it is important to acknowledge certain limitations. Our study had a limited sample size. A larger sample size could provide a more comprehensive understanding of biofilm and resistance patterns. Second, the focus on only two hospitals restricts the diversity of isolates, which might not fully capture the variability in resistance patterns and biofilm production observed in different healthcare settings. Third, the lack of pulsed-field gel electrophoresis (PFGE) or similar genotyping techniques, which provide high-resolution data on genetic relatedness, constrains the ability to determine clonal relationships among isolates. Incorporating these high-resolution methods in future research could offer valuable insights into the genetic mechanisms underlying antibiotic resistance and biofilm formation, thereby enhancing strategies for infection control and antimicrobial stewardship.

The research findings indicate that all *S. aureus* strains isolated from hospital wastewater were MRSA, with a majority exhibiting multidrug resistance. This finding highlights the importance of hospital effluents as sources of clinically relevant bacteria in the environment. It is noteworthy that all MRSA isolates were associated with well-known epidemic clones. Our findings underscore the importance of implementing wastewater treatment systems in hospitals to reduce the dissemination and spread of MRSA strains. The coexistence of resistance and virulence genes in *S. aureus* originating from wastewater carries potential health hazards for both humans and animals. Future studies could include whole-genome sequencing to analyze genetic makeup and virulence factors; investigation the mechanisms of antibiotic resistance; phylogenetic analysis of *S. aureus* strains to understand evolutionary relationships; assessment of biofilm formation capabilities; and study of mobile genetic elements, such as integron, plasmids, and transposons, that may contribute to the spread of antibiotic resistance.

Given the high prevalence of MRSA and VRSA strains identified in this research, along with their biofilm-forming capabilities, targeted interventions are critical. Incorporating advanced wastewater treatment technologies could be recommended to reduce the microbial load and mitigate the dissemination of resistant strains. Additionally, the implementation of routine monitoring programs to assess resistance patterns in hospital effluents would enable early detection and control measures. Strategies such as the use of bacteriophages or antimicrobial coatings in wastewater pipelines could also be explored to inhibit biofilm formation, as biofilm-producing isolates were predominantly observed in this study.

## Figures and Tables

**Table 1:** Distribution of resistance pattern among biofilm producer *S. aureus* isolates.

**Biofilm status**	**Phenotypic resistance (no., %)**	**Number (%)**
Strong producer	PEN, GEN, ERY, CLI, TET (7, 17.9)	39 (55.7)
	PEN, GEN, ERY, NIT, RIF, CLI, TET (2, 5.1)	
	PEN, GEN, TET, RIF, MUP, ERY (3, 7.7)	
	PEN, GEN, CLI, RIF, NIT, TET, MUP (13, 33.3)	
	PEN, GEN, ERY, TET, MUP, NIT, VAN (1, 2.6)	
	PEN, GEN, ERY, TET, MUP, NIT (11, 28.2)	
	PEN, GEN, ERY, CLI, TET, VAN (1, 2.6)	
	PEN, ERY, CLI, TET, NIT (1, 2.6)	

Moderate producer	PEN, GEN, ERY, CLI, TET (12, 54.6)	22 (31.4)
	PEN, ERY, CLI, TET, NIT (5, 22.7)	
	PEN, GEN, TET, RIF, MUP, ERY (2, 9.1)	
	PEN, ERY, CLI, TET, NIT, VAN (1, 4.5)	
	PEN, GEN, ERY, NIT, RIF, CLI, TET (2, 9.1)	

Weak producer	PEN, GEN, ERY, CLI, TET (6, 66.7)	9 (12.9)
	PEN, ERY, CLI, TET, NIT (2, 22.2)	
	PEN, GEN, TET, RIF, MUP, ERY (1, 11.1)	

Abbreviations: CLI, clindamycin; ERY, erythromycin; GEN, gentamicin; MUP, mupirocin; NIT, nitrofurantoin; PEN, penicillin; RIF, rifampicin; TET, tetracycline; VAN, vancomycin.

**Table 2:** Allelic profile of different ST types of *S. aureus* isolates.

**ST**	**arcC**	**aroE**	**glpF**	**gmk**	**pta**	**tpi**	**yqiL**
30	2	2	2	2	6	3	2
22	7	6	1	5	8	8	6
585	2	1	1	1	4	4	3
239	2	3	1	1	4	4	3
8	3	3	1	1	4	4	3
772	1	1	1	1	22	1	1
5	1	4	1	4	12	1	10
1465	2	220	1	1	4	4	3
1	1	1	1	1	1	1	1
421	12	1	1	2	11	1	40
225	1	4	1	4	12	25	10
36	2	2	2	2	3	3	2
1637	1	4	1	127	12	1	10

## Data Availability

The data used to support the findings of this study are included within the article.

## Supporting Information

Supporting Information Additional supporting information can be found online in the Supporting Information section. This supporting information provides detailed nucleotide sequence data for the alleles of the seven housekeeping genes analyzed in the MLST study of *S. aureus* isolates. The sequences serve as a reference for allele identification and facilitate a deeper understanding of genetic variations among the isolates.
