# Supplementary material for: Unveiling the Genetic Landscape of Staphylococcus aureus Isolated From Hospital Wastewaters: Emergence of Hypervirulent CC8 Strains in Tehran, Iran
Source: Int J Microbiol. 2025 Mar 13;2025:5458315. doi: 10.1155/ijm/5458315 (PMC11925629; doi:10.1155/ijm/5458315)
Supplement: Supporting Information — Additional supporting information can be found online in the Supporting Information section. This supporting information provides detailed nucleotide sequence data for the alleles of the seven housekeeping genes analyzed in the MLST study of S. aureus isolates. The sequences serve as a reference for allele identification and facilitate a deeper understanding of genetic variations among the isolates. [file 5458315.f1.docx]

Table S1. Nucleotide sequence among alleles of 7 MLST housekeeping genes of *S. aureus* isolates

| **Sequence type** | **Nucleotide sequence** |
| --- | --- |
| **30** | **>arcC_2**  TTATTAATCCAACAAGCTAAATCGAACAGTGACACAACGCCGGCAATGCCATTGGATACTTGTGGTGCAATGTCACAAGGTATGATAGGCTATTGGTTGGAAACTGAAATCAATCGCATTTTAACTGAAATGAATAGTGATAGAACTGTAGGCACAATCGTAACACGTGTGGAAGTAGATAAAGATGATCCACGATTTGATAACCCAACTAAACCAATTGGTCCTTTTTATACGAAAGAAGAAGTTGAAGAATTACAAAAAGAACAGCCAGGCTCAGTCTTTAAAGAAGATGCAGGACGTGGTTATAGAAAAGTAGTTGCGTCACCACTACCTCAATCTATACTAGAACACCAGTTAATTCGAACTTTAGCAGACGGTAAAAATATTGTCATTGCATGCGGTGGTGGCGGTATTCCAGTTATAAAAAAAGAAAATACCTATGAAGGTGTTGAAGCG  **>aroE_2**  AATTTTAATTCTTTAGGATTAGATGATACTTATGAAGCTTTAAATATTCCAATTGAAGATTTTCATTTAATTAAAGAAATTATTTCAAAAAAAGAATTAGATGGCTTTAATATCACAATTCCTCATAAAGAGCGTATCATACCGTATTTAGATCATGTTGATGAACAAGCGATTAATGCAGGTGCAGTTAACACTGTTTTGATAAAAGATGGCAAGTGGATAGGGTATAATACAGATGGTATTGGTTATGTTAAAGGATTGCACAGCGTTTATCCAGATTTAGAAAATGCATACATTTTAATTTTGGGAGCAGGTGGTGCAAGTAAAGGTATTGCTTATGAATTAGCAAAATTTGTAAAGCCCAAATTAACTGTTGCGAATAGAACGATGGCTCGTTTTGAATCTTGGAATTTAAATATAAACCAAATTTCATTGGCAGATGCTGAAAAGTATTTA  **>glpF_2**  GGTGCTGATTGGATTGTCATCACAGCTGGATGGGGATTAGCGGTTACAATGGGTGTGTATGCTGTTGGTCAATTCTCAGGTGCACATTTAAACCCAGCGGTGTCTTTAGCTCTTGCATTAGACGGAAGTTTTGATTGGTCATTAGTTCCTGGTTATATTGTTGCTCAAATGTTAGGTGCAATTGTCGGAGCAACAATTGTATGGTTAATGTACTTGCCACATTGGAAAGCGACAGAAGAAGCTGGCGCGAAATTAGGTGTTTTCTCTACAGCACCGGCTATTAAGAATTACTTTGCCAACTTTTTAAGTGAAATTATCGGAACAATGGCATTAACTTTAGGTATTTTATTTATCGGTGTAAACAAAATTGCTGATGGTTTAAATCCTTTAATTGTCGGAGCATTAATTGTTGCAATCGGATTAAGTTTAGGCGGTGCTACTGGTTATGCAATCAACCCAGCACGT  **>gmk_2**  CGAATATTTGAAGATCCAAGTACATCATATAAGTATTCTATTTCAATGACAACACGTCAAATGCGTGAAGGTGAAGTTGATGGCGTAGATTACTTTTTTAAAACTAGGGATGCGTTTGAAGCTTTAATTAAAGATGACCAATTTATAGAATATGCTGAATATGTAGGCAACTATTATGGTACACCAGTTCAATATGTTAAAGATACAATGGACGAAGGTCATGATGTATTTTTAGAAATTGAAGTAGAAGGTGCAAAGCAAGTTAGAAAGAAATTTCCAGATGCGTTATTTATTTTCTTAGCACCTCCAAGTTTAGATCACTTGAGAGAGCGATTAGTAGGTAGAGGAACAGAATCTGATGAGAAAATACAAAGTCGTATTAACGAAGCACGTAAAGAAGTCGAAATGATGAATTTA  **>pta_6**  GCAACACAATTACAAGCAACAGATTATGTTACACCAATCGTGTTAGGTGATGAGACTAAGGTTCAATCTTTAGCGCAAAAACTTAATCTTGATATTTCTAATATTGAATTAATTAATCCTGCGACAAGTGAATTGAAAGCTGAATTAGTTCAATCATTTGTTGAACGACGTAAAGGTAAAGCGACTGAAGAACAAGCACAAGAATTATTAAACAATGTGAACTACTTCGGTACAATGCTTGTTTATGCTGGTAAAGCAGATGGTTTAGTTAGTGGTGCAGCACATTCAACAGGCGACACTGTGCGTCCAGCTTTACAAATCATCAAAACGAAACCAGGTGTATCAAGAACATCAGGTATCTTCTTTATGATTAAAGGTGATGAACAGTACATCTTTGGTGATTGTGCAATCAATCCAGAACTTGATTCACAAGGACTTGCAGAAATTGCAGTAGAAAGTGCAAAATCAGCATTA  **>tpi_3**  CACGAAACAGATGAAGAAATTAACAAAAAAGCGCACGCTATTTTCAAACATGGAATGACTCCAATTATTTGTGTTGGTGAAACAGACGAAGAGCGTGAAAGTGGTAAAGCTAACGATGTTGTAGGTGAGCAAGTTAAGAAAGCTGTTGCAGGTTTATCTGAAGATCAACTTAAATCAGTTGTAATTGCTTATGAACCAATCTGGGCAATCGGAACTGGTAAATCATCAACATCTGAAGATGCGAATGAAATGTGTGCATTTGTACGTCAAACTATTGCTGACTTATCAAGCAAAGAAGTATCAGAAGCAACTCGTATTCAATATGGTGGTAGTGTTAAACCTAACAACATTAAAGAATACATGGCACAAACTGATATTGATGGGGCATTAGTAGGTGGCGCA  **>yqiL_2**  GCGTTTAAAGACGTGCCAGCCTATGATTTAGGTGCGACTTTAATAGAACATATTATTAAAGAGACGGGTTTGAATCCAAGTGAGATTAATGAAGTCATCATCGGTAACGTACTACAAGCAGGACAAGGACAAAATCCAGCACGAATTGCTGCTATGAAAGGTGGCTTGCCAGAAACAGTACCTGCATTTACAGTGAATAAAGTATGTGGTTCTGGGTTAAAGTCGATTCAATTAGCATATCAATCTATTGTGACTGGTGAAAATGACATCGTGCTAGCTGGCGGTATGGAGAATATGTCTCAATCACCAATGCTTGTCAACAACAGTCGCTTTGGTTTTAAAATGGGACATCAATCAATGGTTGATAGCATGGTATATGATGGTTTAACAGATGTATTTAATCAATATCATATGGGTATTACTGCTGAAAATTTAGTAGAGCAATATGGTATTTCAAGAGAAGAACAAGATACATTTGCTGTAAACTCACAACAAAAAGCAGTACGTGCACAGCAA |
| **22** | **>arcC_7**  TTATTAATCCAACAAGCTAAATCGAACAGTGACACAACGCCGGCAATGCCATTGGATACTTGTGGTGCAATGTCACAGGGTATGATAGGCTATTGGTTGGAAACTGAAATCAATCGCATTTTAACTGAAATGAATAGTGATAGAACTGTAGGCACAATCGTTACACGTGTGGAAGTAGATAAAGATGATCCACGATTCAATAACCCAACCAAACCAATTGGTCCTTTTTATACGAAAGAAGAAGTTGAAGAATTACAAAAAGAACAGCCAGACTCAGTCTTTAAAGAAGATGCAGGACGTGGTTATAGAAAAGTTGTTGCGTCACCACTACCTCAATCTATACTAGAACACCAGTTAATTCGAACTTTAGCAGACGGTAAAAATATTGTCATTGCATGCGGTGGTGGCGGTATTCCAGTTATAAAAAAAGAAAATACCTATGAAGGTGTTGAAGCG  **>aroE_6**  AATTTTAATTCTTTAGGATTAGATGATACTTATGAAGCTTTAAATATTCCAATTGAAGATTTTCATTTAATTAAAGAAATTATTTCGAAAAAAGAATTAGATGGCTTTAATATCACAATTCCTCATAAAGAGCGTATCATACCGTATTTAGATCATGTTGATGAACAAGCGATTAATGCAGGTGCAGTTAACACTGTTTTGATAAAAGATGGCAAGTGGATAGGGTATAATACAGATGGTATTGGTTATGTTAAAGGATTGCACAGCGTTTATCCAGATTTAGAAAATGCATACATTTTAATTTTGGGCGCAGGTGGTGCAAGTAAAGGTATTGCTTATGAATTAGCAAAATTTGTAAAGCCCAAATTAACTGTTGCGAATAGAACGATGGCTCGTTTTGAATCTTGGAATTTAAATATAAACCAAATTTCAATAGCAGATGCTGAAAAGTATTTA  **>glpF_1**  GGTGCTGATTGGATTGTCATCACAGCTGGATGGGGATTAGCGGTTACAATGGGTGTGTTTGCTGTCGGTCAATTCTCAGGTGCACATTTAAACCCAGCGGTGTCTTTAGCTCTTGCATTAGACGGAAGTTTTGATTGGTCATTAGTTCCTGGTTATATTGTTGCTCAAATGTTAGGTGCAATTGTCGGAGCAACAATTGTATGGTTAATGTACTTGCCACATTGGAAAGCGACAGAAGAAGCTGGCGCGAAATTAGGTGTTTTCTCTACAGCACCGGCTATTAAGAATTACTTTGCCAACTTTTTAAGTGAGATTATCGGAACAATGGCATTAACTTTAGGTATTTTATTTATCGGTGTAAACAAAATTGCCGATGGTTTAAATCCTTTAATTGTCGGAGCATTAATTGTTGCAATCGGATTAAGTTTAGGCGGTGCTACTGGTTATGCAATCAACCCAGCACGT  **>gmk_5**  CGAATATTTGAAGATCCAAGTACATCATATAAGTATTCTATTTCAATGACAACACGTCAAATGCGTGAAGGTGAAGTTGATGGCGTAGATTACTTTTTTAAAACTAGGGATGCGTTTGAAGCTTTAATCAAAGATGACCAATTTATAGAATATGCTGAATATGTAGGCAACTATTATGGTACACCAGTTCAATATGTTAAAGATACAATGGACGAAGGTCATGATGTATTTTTAGAAATTGAAGTAGAAGGTGCAAAGCAAGTTAGAAAGAAATTTCCAGATGCATTATTTATTTTCTTAGCACCTCCAAGTTTAGATCACTTGAGAGAGCGATTAGTAGGTAGAGGAACAGAATCCGATGAGAAAATACAAAGTCGTATTAACGAAGCACGTAAAGAAGTCGAAATGATGAATTTA  **>pta_8**  GCAACACAATTACAAGCAACAGATTATGTTACACCAATCGTGTTAGGTGATGAGACTAAGGTTCAATCTTTAGCGCAAAAACTTAATCTTGATATTTCTAATATTGAATTAATTAATCCTGCGACAAGTGAATTTAAAGCTGAATTAGTCCAATCATTTGTTGAACGACGTAAAGGTAAAGCGACTGAAGAACAAGCGCAAGAATTATTAAACAATGTGAACTACTTCGGTACAATGCTTGTTTATGCTGGTAAAGCAGATGGTTTAGTTAGTGGTGCAGCACATTCAACAGGAGACACTGTGCGTCCAGCTTTACAAATCATCAAAACGAAACCAGGTGTATCAAGAACATCAGGTATCTTCTTTATGATTAAAGGTGATGAACAATACATCTTTGGTGATTGTGCAATCAATCCAGAACTTGATTCACAAGGACTTGCAGAAATTGCAGTAGAAAGTGCAAAATCAGCATTA  **>tpi_8**  CACGAAACAGATAAAGAAATTAACAAAAAAGCGCACGCTATTTTCAAACATGGAATGACTCCAATTATATGTGTTGGTGAAACAGACGAAGAGCGTGAAAGTGGTAAAGCTAACGATGTTGTAGGTGAGCAAGTTAAGAAAGCTGTTGCAGGTTTATCTGAAAATCAACTAAAATCAGTTGTAATTGCTTATGAACCAATCTGGGCAATCGGAACTGGTAAATCATCAACATCTGAAGATGCGAATGAAATGTGTGCATTTGTACGTCAAACTATTGCTGACTTATCAAGCAAAGAAGTATCAGAAGCAACTCGTATTCAATATGGTGGTAGTGTTAAACCTAACAACATTAAAGAATACATGGCACAAACTGATATTGATGGGGCATTAGTAGGTGGCGCA  **>yqiL_6**  GCGTTTAAAGACGTGCCAGCCTATGATTTAGGTGCGACTTTAATAGAACATATTATTAAAGAGACGGGTTTGAATCCAAGTGAGATTGATGAAGTTATCATCGGTAACGTACTACAAGCAGGACAAGGACAAAATCCAGCACGAATTGCTGCTATGAAAGGTGGCTTACCAGAGACAGTACCTGCATTTACAGTGAATAAAGTATGTGGTTCTGGGTTAAAGTCGATTCAATTAGCATATCAATCTATTGTGACTGGTGAAAATGACATCGTGCTAGCTGGCGGTATGGAGAATATGTCTCAATCACCAATGCTTGTCAACAACAGTCGCTTTGGTTTTAAAATGGGACATCAATCAATGGTTGATAGCATGGTATATGATGGTTTAACAGATGTATTTAATCAATATCATATGGGTATTACTGCTGAAAATTTAGTAGAGCAATATGGTATTTCAAGAGAAGAACAAGATACATTTGCTGTAAACTCACAACAAAAAGCAGTACGTGCACAGCAA |
| **585** | **>arcC_2**  TTATTAATCCAACAAGCTAAATCGAACAGTGACACAACGCCGGCAATGCCATTGGATACTTGTGGTGCAATGTCACAAGGTATGATAGGCTATTGGTTGGAAACTGAAATCAATCGCATTTTAACTGAAATGAATAGTGATAGAACTGTAGGCACAATCGTAACACGTGTGGAAGTAGATAAAGATGATCCACGATTTGATAACCCAACTAAACCAATTGGTCCTTTTTATACGAAAGAAGAAGTTGAAGAATTACAAAAAGAACAGCCAGGCTCAGTCTTTAAAGAAGATGCAGGACGTGGTTATAGAAAAGTAGTTGCGTCACCACTACCTCAATCTATACTAGAACACCAGTTAATTCGAACTTTAGCAGACGGTAAAAATATTGTCATTGCATGCGGTGGTGGCGGTATTCCAGTTATAAAAAAAGAAAATACCTATGAAGGTGTTGAAGCG  **>aroE_1**  AATTTTAATTCTTTAGGATTAGATGATACTTATGAAGCTTTAAATATTCCAATTGAAGATTTTCATTTAATTAAAGAAATTATTTCGAAAAAAGAATTAGATGGCTTTAATATCACAATTCCTCATAAAGAACGTATCATACCGTATTTAGATCATGTTGATGAACAAGCGATTAATGCAGGTGCAGTTAACACTGTTTTGATAAAAGATGACAAGTGGATAGGGTATAATACAGATGGTATTGGTTATGTTAAAGGATTGCACAGCGTTTATCCAGATTTAGAAAATGCATACATTTTAATTTTGGGCGCAGGTGGTGCAAGTAAAGGTATTGCTTATGAATTAGCAAAATTTGTAAAGCCCAAATTAACTGTTGCGAATAGAACGATGGCTCGTTTTGAATCTTGGAATTTAAATATAAACCAAATTTCATTAGCAGATGCTGAAAAGTATTTA  **>glpF_1**  GGTGCTGATTGGATTGTCATCACAGCTGGATGGGGATTAGCGGTTACAATGGGTGTGTTTGCTGTCGGTCAATTCTCAGGTGCACATTTAAACCCAGCGGTGTCTTTAGCTCTTGCATTAGACGGAAGTTTTGATTGGTCATTAGTTCCTGGTTATATTGTTGCTCAAATGTTAGGTGCAATTGTCGGAGCAACAATTGTATGGTTAATGTACTTGCCACATTGGAAAGCGACAGAAGAAGCTGGCGCGAAATTAGGTGTTTTCTCTACAGCACCGGCTATTAAGAATTACTTTGCCAACTTTTTAAGTGAGATTATCGGAACAATGGCATTAACTTTAGGTATTTTATTTATCGGTGTAAACAAAATTGCCGATGGTTTAAATCCTTTAATTGTCGGAGCATTAATTGTTGCAATCGGATTAAGTTTAGGCGGTGCTACTGGTTATGCAATCAACCCAGCACGT  **>gmk_1**  CGAATATTTGAAGATCCAAGTACATCATATAAGTATTCTATTTCAATGACAACACGTCAAATGCGTGAAGGTGAAGTTGATGGCGTAGATTACTTTTTTAAAACTAGGGATGCGTTTGAAGCTTTAATCAAAGATGACCAATTTATAGAATATGCTGAATATGTAGGCAACTATTATGGTACACCAGTTCAATATGTTAAAGATACAATGGACGAAGGTCATGATGTATTTTTAGAAATTGAAGTAGAAGGTGCAAAGCAAGTTAGAAAGAAATTTCCAGATGCGCTATTTATTTTCTTAGCACCTCCAAGTTTAGAACACTTGAGAGAGCGATTAGTAGGTAGAGGAACAGAATCTGATGAGAAAATACAAAGTCGTATTAACGAAGCGCGTAAAGAAGTTGAAATGATGAATTTA  **>pta_4**  GCAACACAATTACAAGCAACAGATTATGTTACACCAATCGTGTTAGGTGATGAGACTAAGGTTCAATCTTTAGCGCAAAAACTTGATCTTGATATTTCTAATATTGAATTAATTAATCCTGCGACAAGTGAATTGAAAGCTGAATTAGTTCAATCATTTGTTGAACGACGTAAAGGTAAAGCGACTGAAGAACAAGCACAAGAATTATTAAACAATGTGAACTACTTCGGTACAATGCTTGTTTATGCTGGTAAAGCAGATGGTTTAGTTAGTGGTGCAGCACATTCAACAGGCGACACTGTGCGTCCAGCTTTACAAATCATCAAAACGAAACCAGGTGTATCAAGAACATCAGGTATCTTCTTTATGATTAAAGGTGATGAACAATACATCTTTGGTGATTGTGCAATCAATCCAGAACTTGATTCACAAGGACTTGCAGAAATTGCAGTAGAAAGTGCAAAATCAGCATTA  **>tpi_4**  CACGAAACAGATGAAGAAATTAACAAAAAAGCGCACGCTATTTTCAAACATGGAATGACTCCAATTATATGTGTTGGTGAAACAGACGAAGAGCGTGAAAGTGGTAAAGCTAACGATGTTGTAGGTGAGCAAGTTAAGAAAGCTGTTGCAGGTTTATCTGAAGATCAACTTAAATCAGTTGTAATTGCTTATGAACCAATCTGGGCAATCGGAACTGGTAAATCATCAACATCTGAAGATGCAAATGAAATGTGTGCATTTGTACGTCAAACTATTGCTGACTTATCAAGCAAAGAAGTATCAGAAGCAACTCGTATTCAATATGGTGGTAGTGTTAAACCTAACAACATTAAAGAATACATGGCACAAACTGATATTGATGGGGCATTAGTAGGTGGCGCA  **>yqiL_3**  GCGTTTAAAGACGTGCCAGCCTATGATTTAGGTGCGACTTTAATAGAACATATTATTAAAGAGACGGGTTTGAATCCAAGTGAGATTGATGAAGTTATCATCGGTAACGTACTACAAGCAGGACAAGGACAAAATCCAGCACGAATTGCTGCTATGAAAGGTGGCTTGCCAGAAACAGTACCTGCATTTACGGTGAATAAAGTATGTGGTTCTGGGTTAAAGTCGATTCAATTAGCATATCAATCTATTGTGACTGGTGAAAATGACATCGTGCTAGCTGGCGGTATGGAGAATATGTCTCAATCACCAATGCTTGTCAACAACAGTCGCTTTGGTTTTAAAATGGGACATCAATCAATGGTTGATAGCATGGTATATGATGGTTTAACAGATGTATTTAATCAATATCATATGGGTATTACTGCTGAAAATTTAGTAGAGCAATATGGTATTTCAAGAGAAGAACAAGATACATTTGCTGTAAACTCACAACAAAAAGCAGTACGTGCACAGCAA |
| **239** | **>arcC_2**  TTATTAATCCAACAAGCTAAATCGAACAGTGACACAACGCCGGCAATGCCATTGGATACTTGTGGTGCAATGTCACAAGGTATGATAGGCTATTGGTTGGAAACTGAAATCAATCGCATTTTAACTGAAATGAATAGTGATAGAACTGTAGGCACAATCGTAACACGTGTGGAAGTAGATAAAGATGATCCACGATTTGATAACCCAACTAAACCAATTGGTCCTTTTTATACGAAAGAAGAAGTTGAAGAATTACAAAAAGAACAGCCAGGCTCAGTCTTTAAAGAAGATGCAGGACGTGGTTATAGAAAAGTAGTTGCGTCACCACTACCTCAATCTATACTAGAACACCAGTTAATTCGAACTTTAGCAGACGGTAAAAATATTGTCATTGCATGCGGTGGTGGCGGTATTCCAGTTATAAAAAAAGAAAATACCTATGAAGGTGTTGAAGCG  **>aroE_3**  AATTTTAATTCTTTAGGATTAGATGATACTTATGAAGCTTTAAATATTCCAATTGAAGATTTTCATTTAATTAAAGAAATTATTTCGAAAAAAGAATTAGAAGGCTTTAATATCACAATTCCTCATAAAGAACGTATCATACCGTATTTAGATTATGTTGATGAACAAGCGATTAATGCAGGTGCAGTTAACACTGTTTTGATAAAAGATGGCAAGTGGATAGGGTATAATACAGATGGTATTGGTTATGTTAAAGGATTGCACAGCGTTTATCCAGATTTAGAAAATGCATACATTTTAATTTTGGGCGCAGGTGGTGCAAGTAAAGGTATTGCTTATGAATTAGCAAAATTTGTAAAGCCCAAATTAACTGTTGCGAATAGAACGATGGCTCGTTTTGAATCTTGGAATTTAAATATAAACCAAATTTCATTAGCAGATGCTGAAAAGTATTTA  **>glpF_1**  GGTGCTGATTGGATTGTCATCACAGCTGGATGGGGATTAGCGGTTACAATGGGTGTGTTTGCTGTCGGTCAATTCTCAGGTGCACATTTAAACCCAGCGGTGTCTTTAGCTCTTGCATTAGACGGAAGTTTTGATTGGTCATTAGTTCCTGGTTATATTGTTGCTCAAATGTTAGGTGCAATTGTCGGAGCAACAATTGTATGGTTAATGTACTTGCCACATTGGAAAGCGACAGAAGAAGCTGGCGCGAAATTAGGTGTTTTCTCTACAGCACCGGCTATTAAGAATTACTTTGCCAACTTTTTAAGTGAGATTATCGGAACAATGGCATTAACTTTAGGTATTTTATTTATCGGTGTAAACAAAATTGCCGATGGTTTAAATCCTTTAATTGTCGGAGCATTAATTGTTGCAATCGGATTAAGTTTAGGCGGTGCTACTGGTTATGCAATCAACCCAGCACGT  **>gmk_1**  CGAATATTTGAAGATCCAAGTACATCATATAAGTATTCTATTTCAATGACAACACGTCAAATGCGTGAAGGTGAAGTTGATGGCGTAGATTACTTTTTTAAAACTAGGGATGCGTTTGAAGCTTTAATCAAAGATGACCAATTTATAGAATATGCTGAATATGTAGGCAACTATTATGGTACACCAGTTCAATATGTTAAAGATACAATGGACGAAGGTCATGATGTATTTTTAGAAATTGAAGTAGAAGGTGCAAAGCAAGTTAGAAAGAAATTTCCAGATGCGCTATTTATTTTCTTAGCACCTCCAAGTTTAGAACACTTGAGAGAGCGATTAGTAGGTAGAGGAACAGAATCTGATGAGAAAATACAAAGTCGTATTAACGAAGCGCGTAAAGAAGTTGAAATGATGAATTTA  **>pta_4**  GCAACACAATTACAAGCAACAGATTATGTTACACCAATCGTGTTAGGTGATGAGACTAAGGTTCAATCTTTAGCGCAAAAACTTGATCTTGATATTTCTAATATTGAATTAATTAATCCTGCGACAAGTGAATTGAAAGCTGAATTAGTTCAATCATTTGTTGAACGACGTAAAGGTAAAGCGACTGAAGAACAAGCACAAGAATTATTAAACAATGTGAACTACTTCGGTACAATGCTTGTTTATGCTGGTAAAGCAGATGGTTTAGTTAGTGGTGCAGCACATTCAACAGGCGACACTGTGCGTCCAGCTTTACAAATCATCAAAACGAAACCAGGTGTATCAAGAACATCAGGTATCTTCTTTATGATTAAAGGTGATGAACAATACATCTTTGGTGATTGTGCAATCAATCCAGAACTTGATTCACAAGGACTTGCAGAAATTGCAGTAGAAAGTGCAAAATCAGCATTA  **>tpi_4**  CACGAAACAGATGAAGAAATTAACAAAAAAGCGCACGCTATTTTCAAACATGGAATGACTCCAATTATATGTGTTGGTGAAACAGACGAAGAGCGTGAAAGTGGTAAAGCTAACGATGTTGTAGGTGAGCAAGTTAAGAAAGCTGTTGCAGGTTTATCTGAAGATCAACTTAAATCAGTTGTAATTGCTTATGAACCAATCTGGGCAATCGGAACTGGTAAATCATCAACATCTGAAGATGCAAATGAAATGTGTGCATTTGTACGTCAAACTATTGCTGACTTATCAAGCAAAGAAGTATCAGAAGCAACTCGTATTCAATATGGTGGTAGTGTTAAACCTAACAACATTAAAGAATACATGGCACAAACTGATATTGATGGGGCATTAGTAGGTGGCGCA  **>yqiL_3**  GCGTTTAAAGACGTGCCAGCCTATGATTTAGGTGCGACTTTAATAGAACATATTATTAAAGAGACGGGTTTGAATCCAAGTGAGATTGATGAAGTTATCATCGGTAACGTACTACAAGCAGGACAAGGACAAAATCCAGCACGAATTGCTGCTATGAAAGGTGGCTTGCCAGAAACAGTACCTGCATTTACGGTGAATAAAGTATGTGGTTCTGGGTTAAAGTCGATTCAATTAGCATATCAATCTATTGTGACTGGTGAAAATGACATCGTGCTAGCTGGCGGTATGGAGAATATGTCTCAATCACCAATGCTTGTCAACAACAGTCGCTTTGGTTTTAAAATGGGACATCAATCAATGGTTGATAGCATGGTATATGATGGTTTAACAGATGTATTTAATCAATATCATATGGGTATTACTGCTGAAAATTTAGTAGAGCAATATGGTATTTCAAGAGAAGAACAAGATACATTTGCTGTAAACTCACAACAAAAAGCAGTACGTGCACAGCAA |
| **8** | **>arcC_3**  TTATTAATCCAACAAGCTAAATCGAACAGTGACACAACGCCGGCAATGCCATTGGATACTTGTGGTGCAATGTCACAGGGTATGATAGGCTATTGGTTGGAAACTGAAATCAATCGCATTTTAACTGAAATGAATAGTGATAGAACTGTAGGCACAATCGTTACACGTGTGGAAGTAGATAAAGATGATCCACGATTTGATAACCCAACTAAACCAATTGGTCCTTTTTATACGAAAGAAGAAGTTGAAGAATTACAAAAAGAACAGCCAGACTCAGTCTTTAAAGAAGATGCAGGACGTGGTTATAGAAAAGTAGTTGCGTCACCACTACCTCAATCTATACTAGAACACCAGTTAATTCGAACTTTAGCAGACGGTAAAAATATTGTCATTGCATGCGGTGGTGGCGGTATTCCAGTTATAAAAAAAGAAAATACCTATGAAGGTGTTGAAGCG  **>aroE_3**  AATTTTAATTCTTTAGGATTAGATGATACTTATGAAGCTTTAAATATTCCAATTGAAGATTTTCATTTAATTAAAGAAATTATTTCGAAAAAAGAATTAGAAGGCTTTAATATCACAATTCCTCATAAAGAACGTATCATACCGTATTTAGATTATGTTGATGAACAAGCGATTAATGCAGGTGCAGTTAACACTGTTTTGATAAAAGATGGCAAGTGGATAGGGTATAATACAGATGGTATTGGTTATGTTAAAGGATTGCACAGCGTTTATCCAGATTTAGAAAATGCATACATTTTAATTTTGGGCGCAGGTGGTGCAAGTAAAGGTATTGCTTATGAATTAGCAAAATTTGTAAAGCCCAAATTAACTGTTGCGAATAGAACGATGGCTCGTTTTGAATCTTGGAATTTAAATATAAACCAAATTTCATTAGCAGATGCTGAAAAGTATTTA  **>glpF_1**  GGTGCTGATTGGATTGTCATCACAGCTGGATGGGGATTAGCGGTTACAATGGGTGTGTTTGCTGTCGGTCAATTCTCAGGTGCACATTTAAACCCAGCGGTGTCTTTAGCTCTTGCATTAGACGGAAGTTTTGATTGGTCATTAGTTCCTGGTTATATTGTTGCTCAAATGTTAGGTGCAATTGTCGGAGCAACAATTGTATGGTTAATGTACTTGCCACATTGGAAAGCGACAGAAGAAGCTGGCGCGAAATTAGGTGTTTTCTCTACAGCACCGGCTATTAAGAATTACTTTGCCAACTTTTTAAGTGAGATTATCGGAACAATGGCATTAACTTTAGGTATTTTATTTATCGGTGTAAACAAAATTGCCGATGGTTTAAATCCTTTAATTGTCGGAGCATTAATTGTTGCAATCGGATTAAGTTTAGGCGGTGCTACTGGTTATGCAATCAACCCAGCACGT  **>gmk_1**  CGAATATTTGAAGATCCAAGTACATCATATAAGTATTCTATTTCAATGACAACACGTCAAATGCGTGAAGGTGAAGTTGATGGCGTAGATTACTTTTTTAAAACTAGGGATGCGTTTGAAGCTTTAATCAAAGATGACCAATTTATAGAATATGCTGAATATGTAGGCAACTATTATGGTACACCAGTTCAATATGTTAAAGATACAATGGACGAAGGTCATGATGTATTTTTAGAAATTGAAGTAGAAGGTGCAAAGCAAGTTAGAAAGAAATTTCCAGATGCGCTATTTATTTTCTTAGCACCTCCAAGTTTAGAACACTTGAGAGAGCGATTAGTAGGTAGAGGAACAGAATCTGATGAGAAAATACAAAGTCGTATTAACGAAGCGCGTAAAGAAGTTGAAATGATGAATTTA  **>pta_4**  GCAACACAATTACAAGCAACAGATTATGTTACACCAATCGTGTTAGGTGATGAGACTAAGGTTCAATCTTTAGCGCAAAAACTTGATCTTGATATTTCTAATATTGAATTAATTAATCCTGCGACAAGTGAATTGAAAGCTGAATTAGTTCAATCATTTGTTGAACGACGTAAAGGTAAAGCGACTGAAGAACAAGCACAAGAATTATTAAACAATGTGAACTACTTCGGTACAATGCTTGTTTATGCTGGTAAAGCAGATGGTTTAGTTAGTGGTGCAGCACATTCAACAGGCGACACTGTGCGTCCAGCTTTACAAATCATCAAAACGAAACCAGGTGTATCAAGAACATCAGGTATCTTCTTTATGATTAAAGGTGATGAACAATACATCTTTGGTGATTGTGCAATCAATCCAGAACTTGATTCACAAGGACTTGCAGAAATTGCAGTAGAAAGTGCAAAATCAGCATTA  **>tpi_4**  CACGAAACAGATGAAGAAATTAACAAAAAAGCGCACGCTATTTTCAAACATGGAATGACTCCAATTATATGTGTTGGTGAAACAGACGAAGAGCGTGAAAGTGGTAAAGCTAACGATGTTGTAGGTGAGCAAGTTAAGAAAGCTGTTGCAGGTTTATCTGAAGATCAACTTAAATCAGTTGTAATTGCTTATGAACCAATCTGGGCAATCGGAACTGGTAAATCATCAACATCTGAAGATGCAAATGAAATGTGTGCATTTGTACGTCAAACTATTGCTGACTTATCAAGCAAAGAAGTATCAGAAGCAACTCGTATTCAATATGGTGGTAGTGTTAAACCTAACAACATTAAAGAATACATGGCACAAACTGATATTGATGGGGCATTAGTAGGTGGCGCA  **>yqiL_3**  GCGTTTAAAGACGTGCCAGCCTATGATTTAGGTGCGACTTTAATAGAACATATTATTAAAGAGACGGGTTTGAATCCAAGTGAGATTGATGAAGTTATCATCGGTAACGTACTACAAGCAGGACAAGGACAAAATCCAGCACGAATTGCTGCTATGAAAGGTGGCTTGCCAGAAACAGTACCTGCATTTACGGTGAATAAAGTATGTGGTTCTGGGTTAAAGTCGATTCAATTAGCATATCAATCTATTGTGACTGGTGAAAATGACATCGTGCTAGCTGGCGGTATGGAGAATATGTCTCAATCACCAATGCTTGTCAACAACAGTCGCTTTGGTTTTAAAATGGGACATCAATCAATGGTTGATAGCATGGTATATGATGGTTTAACAGATGTATTTAATCAATATCATATGGGTATTACTGCTGAAAATTTAGTAGAGCAATATGGTATTTCAAGAGAAGAACAAGATACATTTGCTGTAAACTCACAACAAAAAGCAGTACGTGCACAGCAA |
| **772** | **>arcC_1**  TTATTAATCCAACAAGCTAAATCGAACAGTGACACAACGCCGGCAATGCCATTGGATACTTGTGGTGCAATGTCACAGGGTATGATAGGCTATTGGTTGGAAACTGAAATCAATCGCATTTTAACTGAAATGAATAGTGATAGAACTGTAGGCACAATCGTTACACGTGTGGAAGTAGATAAAGATGATCCACGATTCAATAACCCAACCAAACCAATTGGTCCTTTTTATACGAAAGAAGAAGTTGAAGAATTACAAAAAGAACAGCCAGACTCAGTCTTTAAAGAAGATGCAGGACGTGGTTATAGAAAAGTAGTTGCGTCACCACTACCTCAATCTATACTAGAACACCAGTTAATTCGAACTTTAGCAGACGGTAAAAATATTGTCATTGCATGCGGTGGTGGCGGTATTCCAGTTATAAAAAAAGAAAATACCTATGAAGGTGTTGAAGCG  **>aroE_1**  AATTTTAATTCTTTAGGATTAGATGATACTTATGAAGCTTTAAATATTCCAATTGAAGATTTTCATTTAATTAAAGAAATTATTTCGAAAAAAGAATTAGATGGCTTTAATATCACAATTCCTCATAAAGAACGTATCATACCGTATTTAGATCATGTTGATGAACAAGCGATTAATGCAGGTGCAGTTAACACTGTTTTGATAAAAGATGACAAGTGGATAGGGTATAATACAGATGGTATTGGTTATGTTAAAGGATTGCACAGCGTTTATCCAGATTTAGAAAATGCATACATTTTAATTTTGGGCGCAGGTGGTGCAAGTAAAGGTATTGCTTATGAATTAGCAAAATTTGTAAAGCCCAAATTAACTGTTGCGAATAGAACGATGGCTCGTTTTGAATCTTGGAATTTAAATATAAACCAAATTTCATTAGCAGATGCTGAAAAGTATTTA  **>glpF_1**  GGTGCTGATTGGATTGTCATCACAGCTGGATGGGGATTAGCGGTTACAATGGGTGTGTTTGCTGTCGGTCAATTCTCAGGTGCACATTTAAACCCAGCGGTGTCTTTAGCTCTTGCATTAGACGGAAGTTTTGATTGGTCATTAGTTCCTGGTTATATTGTTGCTCAAATGTTAGGTGCAATTGTCGGAGCAACAATTGTATGGTTAATGTACTTGCCACATTGGAAAGCGACAGAAGAAGCTGGCGCGAAATTAGGTGTTTTCTCTACAGCACCGGCTATTAAGAATTACTTTGCCAACTTTTTAAGTGAGATTATCGGAACAATGGCATTAACTTTAGGTATTTTATTTATCGGTGTAAACAAAATTGCCGATGGTTTAAATCCTTTAATTGTCGGAGCATTAATTGTTGCAATCGGATTAAGTTTAGGCGGTGCTACTGGTTATGCAATCAACCCAGCACGT  **>gmk_1**  CGAATATTTGAAGATCCAAGTACATCATATAAGTATTCTATTTCAATGACAACACGTCAAATGCGTGAAGGTGAAGTTGATGGCGTAGATTACTTTTTTAAAACTAGGGATGCGTTTGAAGCTTTAATCAAAGATGACCAATTTATAGAATATGCTGAATATGTAGGCAACTATTATGGTACACCAGTTCAATATGTTAAAGATACAATGGACGAAGGTCATGATGTATTTTTAGAAATTGAAGTAGAAGGTGCAAAGCAAGTTAGAAAGAAATTTCCAGATGCGCTATTTATTTTCTTAGCACCTCCAAGTTTAGAACACTTGAGAGAGCGATTAGTAGGTAGAGGAACAGAATCTGATGAGAAAATACAAAGTCGTATTAACGAAGCGCGTAAAGAAGTTGAAATGATGAATTTA  **>pta_22**  GCAACACAATTACAAGCAACAGATTATGTTACACCAATCGTGTTAGGTGATGAGACTAAGGTTCAATCTTTAGCGCAAAAACTTGATCTTGATATTTCTAATATTGAATTAATTAATCCTGCGACAAGTGAATTGAAAGCTGAATTAGTTCAATCATTTGTTGAACGACGTAAAGGTAAAGCGACTGAAGAACAAGCACAAGAATTATTAAACAATGTGAACTACTTCGGTACAATGCTTGTTTATGCTGGTAAAGCAGATGGTTTAGTTAGTGGTGCAGCACATTCAACAGGAGACACTGTGCGTCCAGCTTTACAAATCATCAAAACGAAACCAGGTGTATCAAGAACATCAGGTATCTTCTTTATGATTAAAGGTGATGAACAATACATCTTTGGCGATTGTGCAATCAATCCAGAACTTGATTCACAAGGACTTGCAGAAATTGCAGTAGAAAGTGCAAAATCAGCATTA  **>tpi_1**  CACGAAACAGATGAAGAAATTAACAAAAAAGCGCACGCTATTTTCAAACATGGAATGACTCCAATTATTTGTGTTGGTGAAACAGACGAAGAGCGTGAAAGTGGTAAAGCTAACGATGTTGTAGGTGAGCAAGTTAAGAAAGCTGTTGCAGGTTTATCTGAAGATCAACTTAAATCAGTTGTAATTGCTTATGAGCCAATCTGGGCAATCGGAACTGGTAAATCATCAACATCTGAAGATGCAAATGAAATGTGTGCATTTGTACGTCAAACTATTGCTGACTTATCAAGCAAAGAAGTATCAGAAGCAACTCGTATTCAATATGGTGGTAGTGTTAAACCTAACAACATTAAAGAATACATGGCACAAACTGATATTGATGGGGCATTAGTAGGTGGCGCA  **>yqiL_1**  GCGTTTAAAGACGTGCCAGCCTATGATTTAGGTGCGACTTTAATAGAACATATTATTAAAGAGACGGGTTTGAATCCAAGTGAGATTGATGAAGTTATCATCGGTAACGTACTACAAGCAGGACAAGGACAAAATCCAGCACGAATTGCTGCTATGAAAGGTGGCTTGCCAGAAACAGTACCTGCATTTACAGTGAATAAAGTATGTGGTTCTGGGTTAAAGTCGATTCAATTAGCATATCAATCTATTGTGACTGGTGAAAATGACATCGTGCTAGCTGGCGGTATGGAGAATATGTCTCAGTCACCAATGCTTGTCAACAACAGTCGCTTCGGTTTTAAAATGGGACATCAATCAATGGTTGATAGCATGGTATATGATGGTTTAACAGATGTATTTAATCAATATCATATGGGTATTACTGCTGAAAATTTAGTGGAGCAATATGGTATTTCAAGAGAAGAACAAGATACATTTGCTGTAAACTCACAACAAAAAGCAGTACGTGCACAGCAA |
| **5** | **>arcC_1**  TTATTAATCCAACAAGCTAAATCGAACAGTGACACAACGCCGGCAATGCCATTGGATACTTGTGGTGCAATGTCACAGGGTATGATAGGCTATTGGTTGGAAACTGAAATCAATCGCATTTTAACTGAAATGAATAGTGATAGAACTGTAGGCACAATCGTTACACGTGTGGAAGTAGATAAAGATGATCCACGATTCAATAACCCAACCAAACCAATTGGTCCTTTTTATACGAAAGAAGAAGTTGAAGAATTACAAAAAGAACAGCCAGACTCAGTCTTTAAAGAAGATGCAGGACGTGGTTATAGAAAAGTAGTTGCGTCACCACTACCTCAATCTATACTAGAACACCAGTTAATTCGAACTTTAGCAGACGGTAAAAATATTGTCATTGCATGCGGTGGTGGCGGTATTCCAGTTATAAAAAAAGAAAATACCTATGAAGGTGTTGAAGCG  **>aroE_4**  AATTTTAATTCTTTAGGATTAGATGATACTTATGAAGCTTTAAATATTCCAATTGAAGATTTTCATTTAATTAAAGAAATTATTTCGAAAAAAGAATTAGATGGCTTTAATATCACAATTCCTCATAAAGAACGTATCATACCGTATTTAGATTATGTTGATGAACAAGCGATTAATGCAGGTGCAGTTAACACTGTTTTGATAAAAGATGGCAAGTGGATAGGGTATAATACAGATGGTATTGGTTATGTTAAAGGATTGCACAGCGTTTATCCAGATTTAGAAAATGCATACATTTTAATTTTGGGCGCAGGTGGTGCAAGTAAAGGTATTGCTTATGAATTAGCAAAATTTGTAAAGCCCAAATTAACTGTTGCGAATAGAACGATGGCTCGTTTTGAATCTTGGAATTTAAATATAAACCAAATTTCATTGGCAGATGCTGAAAAGTATTTA  **>glpF_1**  GGTGCTGATTGGATTGTCATCACAGCTGGATGGGGATTAGCGGTTACAATGGGTGTGTTTGCTGTCGGTCAATTCTCAGGTGCACATTTAAACCCAGCGGTGTCTTTAGCTCTTGCATTAGACGGAAGTTTTGATTGGTCATTAGTTCCTGGTTATATTGTTGCTCAAATGTTAGGTGCAATTGTCGGAGCAACAATTGTATGGTTAATGTACTTGCCACATTGGAAAGCGACAGAAGAAGCTGGCGCGAAATTAGGTGTTTTCTCTACAGCACCGGCTATTAAGAATTACTTTGCCAACTTTTTAAGTGAGATTATCGGAACAATGGCATTAACTTTAGGTATTTTATTTATCGGTGTAAACAAAATTGCCGATGGTTTAAATCCTTTAATTGTCGGAGCATTAATTGTTGCAATCGGATTAAGTTTAGGCGGTGCTACTGGTTATGCAATCAACCCAGCACGT  **>gmk_4**  CGAATATTTGAAGATCCAAGTACATCATATAAGTATTCTATTTCAATGACAACACGTCAAATGCGTGAAGGTGAAGTTGATGGCGTAGATTACTTTTTTAAAACTAGGGATGCGTTTGAAGCTTTAATTAAAGATGACCAATTTATAGAATATGCTGAATATGTAGGCAACTATTATGGTACACCAGTTCAATATGTTAAAGATACAATGGACGAAGGTCATGATGTATTTTTAGAAATTGAAGTAGAAGGTGCAAAGCAAGTTAGAAAGAAATTTCCAGATGCGTTATTTATTTTCTTAGCACCTCCAAGTTTAGATCACTTGAGAGAGCGATTAGTAGGTAGAGGAACAGAATCCAATGAGAAAATACAAAGTCGTATTAACGAAGCGCGTAAAGAAGTTGAAATGATGAATTT  **>pta_12**  GCAACACAATTACAAGCAACAGATTATGTTACACCAATCGTGTTAGGTGATGAGACTAAGGTTCAATCTTTAGCGCAAAAACTTGATCTTGATATTTCTAATATTGAATTAATTAATCCTGCGACAAGTGAATTGAAAGCTGAATTAGTTCAATCATTTGTTGAACGACGTAAAGGTAAAGCGACTGAAGAACAAGCACAAGAATTATTAAACAATGTGAACTACTTCGGTACAATGCTTGTTTATGCTGGTAAAGCAGATGGTTTAGTTAGTGGTGCAGCACATTCAACAGGAGACACTGTGCGTCCAGCTTTACAAATCATCAAAACGAAACCAGGTGTATCAAGAACATCAGGTATCTTCTTTATGATTAAAGGTGATGAACAATACATCTTTGGTGATTGTGCAATCAATCCAGAACTTGATTCACAAGGACTTGCAGAAATTGCAGTAGAAAGTGCAAAATCAGCATTA  **>tpi_1**  CACGAAACAGATGAAGAAATTAACAAAAAAGCGCACGCTATTTTCAAACATGGAATGACTCCAATTATTTGTGTTGGTGAAACAGACGAAGAGCGTGAAAGTGGTAAAGCTAACGATGTTGTAGGTGAGCAAGTTAAGAAAGCTGTTGCAGGTTTATCTGAAGATCAACTTAAATCAGTTGTAATTGCTTATGAGCCAATCTGGGCAATCGGAACTGGTAAATCATCAACATCTGAAGATGCAAATGAAATGTGTGCATTTGTACGTCAAACTATTGCTGACTTATCAAGCAAAGAAGTATCAGAAGCAACTCGTATTCAATATGGTGGTAGTGTTAAACCTAACAACATTAAAGAATACATGGCACAAACTGATATTGATGGGGCATTAGTAGGTGGCGCA  **>yqiL_10**  GCGTTTAAAGACGTGCCAGCCTATGATTTAGGTGCGACTTTAATAGAACATATTATTAAAGAGACGGGTTTGAATCCAAGTGAGATTGATGAAGTTATCATCGGTAACGTACTACAAGCAGGACAAGGACAAAATCCAGCACGAATTGCTGCTATGAAAGGTGGCTTGCCAGAAACAGTACCTGCATTTACAGTGAATAAAGTATGTGGTTCTGGGTTAAAGTCGATTCAATTAGCATATCAATCTATTGTGACTGGTGAAAATGACATCGTGCTAGCTGGCGGTATGGAGAATATGTCTCAGTCACCAATGCTTGTCAACAACAGTCGCTTCGGTTTTAAAATGGGACATCAATCAATGGTTGATAGCATGGTATATGATGGTTTAACAGATGTATTTAATCAATATCATATGGGTATTACTGCTGAAAATTTAGTAGAGCAATATGGTATTTCAAGAGAAGAACAAGATACATTTGCTGTAAACTCACAACATAAAGCAGTACGTGCACAGCAA |
| **1465** | **>arcC_2**  TTATTAATCCAACAAGCTAAATCGAACAGTGACACAACGCCGGCAATGCCATTGGATACTTGTGGTGCAATGTCACAAGGTATGATAGGCTATTGGTTGGAAACTGAAATCAATCGCATTTTAACTGAAATGAATAGTGATAGAACTGTAGGCACAATCGTAACACGTGTGGAAGTAGATAAAGATGATCCACGATTTGATAACCCAACTAAACCAATTGGTCCTTTTTATACGAAAGAAGAAGTTGAAGAATTACAAAAAGAACAGCCAGGCTCAGTCTTTAAAGAAGATGCAGGACGTGGTTATAGAAAAGTAGTTGCGTCACCACTACCTCAATCTATACTAGAACACCAGTTAATTCGAACTTTAGCAGACGGTAAAAATATTGTCATTGCATGCGGTGGTGGCGGTATTCCAGTTATAAAAAAAGAAAATACCTATGAAGGTGTTGAAGCG  **>aroE_220**  AATTTTAATTCTTTAGGATTAGATGATACTTATGAAGCTTTAAATATTCCAATTGAAGATTTTCATTTAATTAAAGAAATTATTTCGAAAAAAGAATTAGAAAGCTTTAATATCACAATTCCTCATAAAGAACGTATCATACCGTATTTAGATTATGTTGATGAACAAGCGATTAATGCAGGTGCAGTTAACACTGTTTTGATAAAAGATGGCAAGTGGATAGGGTATAATACAGATGGTATTGGTTATGTTAAAGGATTGCACAGCGTTTATCCAGATTTAGAAAATGCATACATTTTAATTTTGGGCGCAGGTGGTGCAAGTAAAGGTATTGCTTATGAATTAGCAAAATTTGTAAAGCCCAAATTAACTGTTGCGAATAGAACGATGGCTCGTTTTGAATCTTGGAATTTAAATATAAACCAAATTTCATTAGCAGATGCTGAAAAGTATTTA  **>glpF_1**  GGTGCTGATTGGATTGTCATCACAGCTGGATGGGGATTAGCGGTTACAATGGGTGTGTTTGCTGTCGGTCAATTCTCAGGTGCACATTTAAACCCAGCGGTGTCTTTAGCTCTTGCATTAGACGGAAGTTTTGATTGGTCATTAGTTCCTGGTTATATTGTTGCTCAAATGTTAGGTGCAATTGTCGGAGCAACAATTGTATGGTTAATGTACTTGCCACATTGGAAAGCGACAGAAGAAGCTGGCGCGAAATTAGGTGTTTTCTCTACAGCACCGGCTATTAAGAATTACTTTGCCAACTTTTTAAGTGAGATTATCGGAACAATGGCATTAACTTTAGGTATTTTATTTATCGGTGTAAACAAAATTGCCGATGGTTTAAATCCTTTAATTGTCGGAGCATTAATTGTTGCAATCGGATTAAGTTTAGGCGGTGCTACTGGTTATGCAATCAACCCAGCACGT  **>gmk_1**  CGAATATTTGAAGATCCAAGTACATCATATAAGTATTCTATTTCAATGACAACACGTCAAATGCGTGAAGGTGAAGTTGATGGCGTAGATTACTTTTTTAAAACTAGGGATGCGTTTGAAGCTTTAATCAAAGATGACCAATTTATAGAATATGCTGAATATGTAGGCAACTATTATGGTACACCAGTTCAATATGTTAAAGATACAATGGACGAAGGTCATGATGTATTTTTAGAAATTGAAGTAGAAGGTGCAAAGCAAGTTAGAAAGAAATTTCCAGATGCGCTATTTATTTTCTTAGCACCTCCAAGTTTAGAACACTTGAGAGAGCGATTAGTAGGTAGAGGAACAGAATCTGATGAGAAAATACAAAGTCGTATTAACGAAGCGCGTAAAGAAGTTGAAATGATGAATTTA  **>pta_4**  GCAACACAATTACAAGCAACAGATTATGTTACACCAATCGTGTTAGGTGATGAGACTAAGGTTCAATCTTTAGCGCAAAAACTTGATCTTGATATTTCTAATATTGAATTAATTAATCCTGCGACAAGTGAATTGAAAGCTGAATTAGTTCAATCATTTGTTGAACGACGTAAAGGTAAAGCGACTGAAGAACAAGCACAAGAATTATTAAACAATGTGAACTACTTCGGTACAATGCTTGTTTATGCTGGTAAAGCAGATGGTTTAGTTAGTGGTGCAGCACATTCAACAGGCGACACTGTGCGTCCAGCTTTACAAATCATCAAAACGAAACCAGGTGTATCAAGAACATCAGGTATCTTCTTTATGATTAAAGGTGATGAACAATACATCTTTGGTGATTGTGCAATCAATCCAGAACTTGATTCACAAGGACTTGCAGAAATTGCAGTAGAAAGTGCAAAATCAGCATTA  **>tpi_4**  CACGAAACAGATGAAGAAATTAACAAAAAAGCGCACGCTATTTTCAAACATGGAATGACTCCAATTATATGTGTTGGTGAAACAGACGAAGAGCGTGAAAGTGGTAAAGCTAACGATGTTGTAGGTGAGCAAGTTAAGAAAGCTGTTGCAGGTTTATCTGAAGATCAACTTAAATCAGTTGTAATTGCTTATGAACCAATCTGGGCAATCGGAACTGGTAAATCATCAACATCTGAAGATGCAAATGAAATGTGTGCATTTGTACGTCAAACTATTGCTGACTTATCAAGCAAAGAAGTATCAGAAGCAACTCGTATTCAATATGGTGGTAGTGTTAAACCTAACAACATTAAAGAATACATGGCACAAACTGATATTGATGGGGCATTAGTAGGTGGCGCA  **>yqiL_3**  GCGTTTAAAGACGTGCCAGCCTATGATTTAGGTGCGACTTTAATAGAACATATTATTAAAGAGACGGGTTTGAATCCAAGTGAGATTGATGAAGTTATCATCGGTAACGTACTACAAGCAGGACAAGGACAAAATCCAGCACGAATTGCTGCTATGAAAGGTGGCTTGCCAGAAACAGTACCTGCATTTACGGTGAATAAAGTATGTGGTTCTGGGTTAAAGTCGATTCAATTAGCATATCAATCTATTGTGACTGGTGAAAATGACATCGTGCTAGCTGGCGGTATGGAGAATATGTCTCAATCACCAATGCTTGTCAACAACAGTCGCTTTGGTTTTAAAATGGGACATCAATCAATGGTTGATAGCATGGTATATGATGGTTTAACAGATGTATTTAATCAATATCATATGGGTATTACTGCTGAAAATTTAGTAGAGCAATATGGTATTTCAAGAGAAGAACAAGATACATTTGCTGTAAACTCACAACAAAAAGCAGTACGTGCACAGCAA |
| **1** | **>arcC_1**  TTATTAATCCAACAAGCTAAATCGAACAGTGACACAACGCCGGCAATGCCATTGGATACTTGTGGTGCAATGTCACAGGGTATGATAGGCTATTGGTTGGAAACTGAAATCAATCGCATTTTAACTGAAATGAATAGTGATAGAACTGTAGGCACAATCGTTACACGTGTGGAAGTAGATAAAGATGATCCACGATTCAATAACCCAACCAAACCAATTGGTCCTTTTTATACGAAAGAAGAAGTTGAAGAATTACAAAAAGAACAGCCAGACTCAGTCTTTAAAGAAGATGCAGGACGTGGTTATAGAAAAGTAGTTGCGTCACCACTACCTCAATCTATACTAGAACACCAGTTAATTCGAACTTTAGCAGACGGTAAAAATATTGTCATTGCATGCGGTGGTGGCGGTATTCCAGTTATAAAAAAAGAAAATACCTATGAAGGTGTTGAAGCG  **>aroE_1**  AATTTTAATTCTTTAGGATTAGATGATACTTATGAAGCTTTAAATATTCCAATTGAAGATTTTCATTTAATTAAAGAAATTATTTCGAAAAAAGAATTAGATGGCTTTAATATCACAATTCCTCATAAAGAACGTATCATACCGTATTTAGATCATGTTGATGAACAAGCGATTAATGCAGGTGCAGTTAACACTGTTTTGATAAAAGATGACAAGTGGATAGGGTATAATACAGATGGTATTGGTTATGTTAAAGGATTGCACAGCGTTTATCCAGATTTAGAAAATGCATACATTTTAATTTTGGGCGCAGGTGGTGCAAGTAAAGGTATTGCTTATGAATTAGCAAAATTTGTAAAGCCCAAATTAACTGTTGCGAATAGAACGATGGCTCGTTTTGAATCTTGGAATTTAAATATAAACCAAATTTCATTAGCAGATGCTGAAAAGTATTTA  **>glpF_1**  GGTGCTGATTGGATTGTCATCACAGCTGGATGGGGATTAGCGGTTACAATGGGTGTGTTTGCTGTCGGTCAATTCTCAGGTGCACATTTAAACCCAGCGGTGTCTTTAGCTCTTGCATTAGACGGAAGTTTTGATTGGTCATTAGTTCCTGGTTATATTGTTGCTCAAATGTTAGGTGCAATTGTCGGAGCAACAATTGTATGGTTAATGTACTTGCCACATTGGAAAGCGACAGAAGAAGCTGGCGCGAAATTAGGTGTTTTCTCTACAGCACCGGCTATTAAGAATTACTTTGCCAACTTTTTAAGTGAGATTATCGGAACAATGGCATTAACTTTAGGTATTTTATTTATCGGTGTAAACAAAATTGCCGATGGTTTAAATCCTTTAATTGTCGGAGCATTAATTGTTGCAATCGGATTAAGTTTAGGCGGTGCTACTGGTTATGCAATCAACCCAGCACGT  **>gmk_1**  CGAATATTTGAAGATCCAAGTACATCATATAAGTATTCTATTTCAATGACAACACGTCAAATGCGTGAAGGTGAAGTTGATGGCGTAGATTACTTTTTTAAAACTAGGGATGCGTTTGAAGCTTTAATCAAAGATGACCAATTTATAGAATATGCTGAATATGTAGGCAACTATTATGGTACACCAGTTCAATATGTTAAAGATACAATGGACGAAGGTCATGATGTATTTTTAGAAATTGAAGTAGAAGGTGCAAAGCAAGTTAGAAAGAAATTTCCAGATGCGCTATTTATTTTCTTAGCACCTCCAAGTTTAGAACACTTGAGAGAGCGATTAGTAGGTAGAGGAACAGAATCTGATGAGAAAATACAAAGTCGTATTAACGAAGCGCGTAAAGAAGTTGAAATGATGAATTTA  **>pta_1**  GCAACACAATTACAAGCAACAGATTATGTTACACCAATCGTGTTAGGTGATGAGACTAAGGTTCAATCTTTAGCGCAAAAACTTGATCTTGATATTTCTAATATTGAATTAATTAATCCTGCGACAAGTGAATTGAAAGCTGAATTAGTTCAATCATTTGTTGAACGACGTAAAGGTAAAGCGACTGAAGAACAAGCACAAGAATTATTAAACAATGTGAACTACTTCGGTACAATGCTTGTTTATGCTGGTAAAGCAGATGGTTTAGTTAGTGGTGCAGCACATTCAACAGGAGACACTGTGCGTCCAGCTTTACAAATCATCAAAACGAAACCAGGTGTATCAAGAACATCAGGTATCTTCTTTATGATTAAAGGTGATGTACAATACATCTTTGGTGATTGTGCAATCAATCCAGAACTTGATTCACAAGGACTTGCAGAAATTGCAGTAGAAAGTGCAAAATCAGCATTA  **>tpi_1**  CACGAAACAGATGAAGAAATTAACAAAAAAGCGCACGCTATTTTCAAACATGGAATGACTCCAATTATTTGTGTTGGTGAAACAGACGAAGAGCGTGAAAGTGGTAAAGCTAACGATGTTGTAGGTGAGCAAGTTAAGAAAGCTGTTGCAGGTTTATCTGAAGATCAACTTAAATCAGTTGTAATTGCTTATGAGCCAATCTGGGCAATCGGAACTGGTAAATCATCAACATCTGAAGATGCAAATGAAATGTGTGCATTTGTACGTCAAACTATTGCTGACTTATCAAGCAAAGAAGTATCAGAAGCAACTCGTATTCAATATGGTGGTAGTGTTAAACCTAACAACATTAAAGAATACATGGCACAAACTGATATTGATGGGGCATTAGTAGGTGGCGCA  **>yqiL_1**  GCGTTTAAAGACGTGCCAGCCTATGATTTAGGTGCGACTTTAATAGAACATATTATTAAAGAGACGGGTTTGAATCCAAGTGAGATTGATGAAGTTATCATCGGTAACGTACTACAAGCAGGACAAGGACAAAATCCAGCACGAATTGCTGCTATGAAAGGTGGCTTGCCAGAAACAGTACCTGCATTTACAGTGAATAAAGTATGTGGTTCTGGGTTAAAGTCGATTCAATTAGCATATCAATCTATTGTGACTGGTGAAAATGACATCGTGCTAGCTGGCGGTATGGAGAATATGTCTCAGTCACCAATGCTTGTCAACAACAGTCGCTTCGGTTTTAAAATGGGACATCAATCAATGGTTGATAGCATGGTATATGATGGTTTAACAGATGTATTTAATCAATATCATATGGGTATTACTGCTGAAAATTTAGTGGAGCAATATGGTATTTCAAGAGAAGAACAAGATACATTTGCTGTAAACTCACAACAAAAAGCAGTACGTGCACAGCAA |
| **421** | **>arcC_12**  TTATTAATCCAACAAGCTAAATCGAACAGTGACACAACGCCGGCAATGCCATTGGATACTTGTGGTGCAATGTCACAGGGTATGATAGGCTATTGGTTGGAAACTGAAATCAATCGCATTTTAACTGAAATGAATAGTGATAGAACCGTAGGCACAATCGTTACACGTGTGGAAGTAGATAAAGATGATCCAAGATTTGATAACCCAACTAAACCAATTGGTCCTTTTTATACGAAAGAAGAAGTTGAAGAATTACAAAAAGAACAGCCAGACTCAGTCTTTAAAGAAGATGCAGGACGTGGTTATAGAAAAGTAGTTGCGTCACCACTACCTCAATCTATACTAGAACACCAGTTAATTCGAACTTTAGCAGACGGTAAAAATATTGTCATTGCATGCGGTGGTGGCGGTATTCCAGTTATAAAAAAAGAAAATACCTATGAAGGTGTTGAAGCG  **>aroE_1**  AATTTTAATTCTTTAGGATTAGATGATACTTATGAAGCTTTAAATATTCCAATTGAAGATTTTCATTTAATTAAAGAAATTATTTCGAAAAAAGAATTAGATGGCTTTAATATCACAATTCCTCATAAAGAACGTATCATACCGTATTTAGATCATGTTGATGAACAAGCGATTAATGCAGGTGCAGTTAACACTGTTTTGATAAAAGATGACAAGTGGATAGGGTATAATACAGATGGTATTGGTTATGTTAAAGGATTGCACAGCGTTTATCCAGATTTAGAAAATGCATACATTTTAATTTTGGGCGCAGGTGGTGCAAGTAAAGGTATTGCTTATGAATTAGCAAAATTTGTAAAGCCCAAATTAACTGTTGCGAATAGAACGATGGCTCGTTTTGAATCTTGGAATTTAAATATAAACCAAATTTCATTAGCAGATGCTGAAAAGTATTTA  **>glpF_1**  GGTGCTGATTGGATTGTCATCACAGCTGGATGGGGATTAGCGGTTACAATGGGTGTGTTTGCTGTCGGTCAATTCTCAGGTGCACATTTAAACCCAGCGGTGTCTTTAGCTCTTGCATTAGACGGAAGTTTTGATTGGTCATTAGTTCCTGGTTATATTGTTGCTCAAATGTTAGGTGCAATTGTCGGAGCAACAATTGTATGGTTAATGTACTTGCCACATTGGAAAGCGACAGAAGAAGCTGGCGCGAAATTAGGTGTTTTCTCTACAGCACCGGCTATTAAGAATTACTTTGCCAACTTTTTAAGTGAGATTATCGGAACAATGGCATTAACTTTAGGTATTTTATTTATCGGTGTAAACAAAATTGCCGATGGTTTAAATCCTTTAATTGTCGGAGCATTAATTGTTGCAATCGGATTAAGTTTAGGCGGTGCTACTGGTTATGCAATCAACCCAGCACGT  **>gmk_2**  CGAATATTTGAAGATCCAAGTACATCATATAAGTATTCTATTTCAATGACAACACGTCAAATGCGTGAAGGTGAAGTTGATGGCGTAGATTACTTTTTTAAAACTAGGGATGCGTTTGAAGCTTTAATTAAAGATGACCAATTTATAGAATATGCTGAATATGTAGGCAACTATTATGGTACACCAGTTCAATATGTTAAAGATACAATGGACGAAGGTCATGATGTATTTTTAGAAATTGAAGTAGAAGGTGCAAAGCAAGTTAGAAAGAAATTTCCAGATGCGTTATTTATTTTCTTAGCACCTCCAAGTTTAGATCACTTGAGAGAGCGATTAGTAGGTAGAGGAACAGAATCTGATGAGAAAATACAAAGTCGTATTAACGAAGCACGTAAAGAAGTCGAAATGATGAATTTA  **>pta_11**  GCAACACAATTACAAGCAACAGATTATGTTACACCAATCGTGTTAGGTGATGAGACTAAGGTTCAATCTTTAGCGCAAAAACTTAATCTTGATATTTCTAATATTGAATTAATTAATCCTGCGACAAGTGAATTGAAAGCTGAATTAGTCCAATCATTTGTTGAACGACGTAAAGGTAAAGCGACTGAAGAACAAGCGCAAGAATTATTAAACAATGTGAACTACTTCGGTACAATGCTTGTTTATGCTGGTAAAGCAGATGGTTTAGTTAGTGGTGCAGCACATTCAACAGGCGACACTGTGCGTCCAGCTTTACAAATCATCAAAACGAAACCAGGTGTATCAAGAACATCAGGTATCTTCTTTATGATTAAAGGTGATGAACAATACATCTTTGGTGATTGTGCAATCAATCCAGAACTTGATTCACAAGGACTTGCAGAAATTGCAGTAGAAAGTGCAAAATCAGCATTA  **>tpi_1**  CACGAAACAGATGAAGAAATTAACAAAAAAGCGCACGCTATTTTCAAACATGGAATGACTCCAATTATTTGTGTTGGTGAAACAGACGAAGAGCGTGAAAGTGGTAAAGCTAACGATGTTGTAGGTGAGCAAGTTAAGAAAGCTGTTGCAGGTTTATCTGAAGATCAACTTAAATCAGTTGTAATTGCTTATGAGCCAATCTGGGCAATCGGAACTGGTAAATCATCAACATCTGAAGATGCAAATGAAATGTGTGCATTTGTACGTCAAACTATTGCTGACTTATCAAGCAAAGAAGTATCAGAAGCAACTCGTATTCAATATGGTGGTAGTGTTAAACCTAACAACATTAAAGAATACATGGCACAAACTGATATTGATGGGGCATTAGTAGGTGGCGCA  **>yqiL_40**  GCGTTTAAAGACGTGCCAGCCTATGATTTAGGTGCGACTTTAATAGAACATATTATTAAAGAGACGGGTTTGAATCCAAGTGAGATTGATGAAGTTATCATCGGTAACGTACTACAAGCAGGACAAGGACAAAATCCAGCACGAATTGCTGCTATGAAAGGTGGCTTGCCAGAAACAGTACCTGCATTTACAGTGAATAAAGTATGTGGTTCTGGGTTAAAGTCGATTCAATTAGCATATCAATCTATTGTGACTGGTGAAAATGACATCGTGCTAGCTGGCGGTATGGAGAATATGTCTCAGTCACCAATGCTTGTCAACAACAGTCGCTTCGGTTTTAAAATGGGACATCAATCAATGGTTGATAGCATGGTATATGATGGTTTAACAGATGTATTTAATCAATATCATATGGGTATTACTGCTGAAAATTTAGTAGAGCAATATGGTATTTCAAGAGAAGAACAAGATACATTTGCTGTAAACTCACAACAAAAAGCAGTACGTGCACAGCAA |
| **225** | **>arcC_1**  TTATTAATCCAACAAGCTAAATCGAACAGTGACACAACGCCGGCAATGCCATTGGATACTTGTGGTGCAATGTCACAGGGTATGATAGGCTATTGGTTGGAAACTGAAATCAATCGCATTTTAACTGAAATGAATAGTGATAGAACTGTAGGCACAATCGTTACACGTGTGGAAGTAGATAAAGATGATCCACGATTCAATAACCCAACCAAACCAATTGGTCCTTTTTATACGAAAGAAGAAGTTGAAGAATTACAAAAAGAACAGCCAGACTCAGTCTTTAAAGAAGATGCAGGACGTGGTTATAGAAAAGTAGTTGCGTCACCACTACCTCAATCTATACTAGAACACCAGTTAATTCGAACTTTAGCAGACGGTAAAAATATTGTCATTGCATGCGGTGGTGGCGGTATTCCAGTTATAAAAAAAGAAAATACCTATGAAGGTGTTGAAGCG  **>aroE_4**  AATTTTAATTCTTTAGGATTAGATGATACTTATGAAGCTTTAAATATTCCAATTGAAGATTTTCATTTAATTAAAGAAATTATTTCGAAAAAAGAATTAGATGGCTTTAATATCACAATTCCTCATAAAGAACGTATCATACCGTATTTAGATTATGTTGATGAACAAGCGATTAATGCAGGTGCAGTTAACACTGTTTTGATAAAAGATGGCAAGTGGATAGGGTATAATACAGATGGTATTGGTTATGTTAAAGGATTGCACAGCGTTTATCCAGATTTAGAAAATGCATACATTTTAATTTTGGGCGCAGGTGGTGCAAGTAAAGGTATTGCTTATGAATTAGCAAAATTTGTAAAGCCCAAATTAACTGTTGCGAATAGAACGATGGCTCGTTTTGAATCTTGGAATTTAAATATAAACCAAATTTCATTGGCAGATGCTGAAAAGTATTTA  **>glpF_1**  GGTGCTGATTGGATTGTCATCACAGCTGGATGGGGATTAGCGGTTACAATGGGTGTGTTTGCTGTCGGTCAATTCTCAGGTGCACATTTAAACCCAGCGGTGTCTTTAGCTCTTGCATTAGACGGAAGTTTTGATTGGTCATTAGTTCCTGGTTATATTGTTGCTCAAATGTTAGGTGCAATTGTCGGAGCAACAATTGTATGGTTAATGTACTTGCCACATTGGAAAGCGACAGAAGAAGCTGGCGCGAAATTAGGTGTTTTCTCTACAGCACCGGCTATTAAGAATTACTTTGCCAACTTTTTAAGTGAGATTATCGGAACAATGGCATTAACTTTAGGTATTTTATTTATCGGTGTAAACAAAATTGCCGATGGTTTAAATCCTTTAATTGTCGGAGCATTAATTGTTGCAATCGGATTAAGTTTAGGCGGTGCTACTGGTTATGCAATCAACCCAGCACGT  **>gmk_4**  CGAATATTTGAAGATCCAAGTACATCATATAAGTATTCTATTTCAATGACAACACGTCAAATGCGTGAAGGTGAAGTTGATGGCGTAGATTACTTTTTTAAAACTAGGGATGCGTTTGAAGCTTTAATTAAAGATGACCAATTTATAGAATATGCTGAATATGTAGGCAACTATTATGGTACACCAGTTCAATATGTTAAAGATACAATGGACGAAGGTCATGATGTATTTTTAGAAATTGAAGTAGAAGGTGCAAAGCAAGTTAGAAAGAAATTTCCAGATGCGTTATTTATTTTCTTAGCACCTCCAAGTTTAGATCACTTGAGAGAGCGATTAGTAGGTAGAGGAACAGAATCCAATGAGAAAATACAAAGTCGTATTAACGAAGCGCGTAAAGAAGTTGAAATGATGAATTT  **>pta_12**  GCAACACAATTACAAGCAACAGATTATGTTACACCAATCGTGTTAGGTGATGAGACTAAGGTTCAATCTTTAGCGCAAAAACTTGATCTTGATATTTCTAATATTGAATTAATTAATCCTGCGACAAGTGAATTGAAAGCTGAATTAGTTCAATCATTTGTTGAACGACGTAAAGGTAAAGCGACTGAAGAACAAGCACAAGAATTATTAAACAATGTGAACTACTTCGGTACAATGCTTGTTTATGCTGGTAAAGCAGATGGTTTAGTTAGTGGTGCAGCACATTCAACAGGAGACACTGTGCGTCCAGCTTTACAAATCATCAAAACGAAACCAGGTGTATCAAGAACATCAGGTATCTTCTTTATGATTAAAGGTGATGAACAATACATCTTTGGTGATTGTGCAATCAATCCAGAACTTGATTCACAAGGACTTGCAGAAATTGCAGTAGAAAGTGCAAAATCAGCATTA  **>tpi_25**  CACGAAACAGATGAAGAAATTAACAAAAAAGCGCACGCTATTTTCAAACATGGAATGACTCCAATTATTTGTGTTGGTGAAACAGACGAAGAGCGTGAAAGTGGTAAAGCTAACGATGTTGTAGGTGAGCAAGTTAAGAAAGCTGTTGCAGGCTTATCTGAAGATCAACTTAAATCAGTTGTAATTGCTTATGAGCCAATCTGGGCAATCGGAACTGGTAAATCATCAACATCTGAAGATGCAAATGAAATGTGTGCATTTGTACGTCAAACTATTGCTGACTTATCAAGCAAAGAAGTATCAGAAGCAACTCGTATTCAATATGGTGGTAGTGTTAAACCTAACAACATTAAAGAATACATGGCACAAACTGATATTGATGGGGCATTAGTAGGTGGCGCA  **>yqiL_10**  GCGTTTAAAGACGTGCCAGCCTATGATTTAGGTGCGACTTTAATAGAACATATTATTAAAGAGACGGGTTTGAATCCAAGTGAGATTGATGAAGTTATCATCGGTAACGTACTACAAGCAGGACAAGGACAAAATCCAGCACGAATTGCTGCTATGAAAGGTGGCTTGCCAGAAACAGTACCTGCATTTACAGTGAATAAAGTATGTGGTTCTGGGTTAAAGTCGATTCAATTAGCATATCAATCTATTGTGACTGGTGAAAATGACATCGTGCTAGCTGGCGGTATGGAGAATATGTCTCAGTCACCAATGCTTGTCAACAACAGTCGCTTCGGTTTTAAAATGGGACATCAATCAATGGTTGATAGCATGGTATATGATGGTTTAACAGATGTATTTAATCAATATCATATGGGTATTACTGCTGAAAATTTAGTAGAGCAATATGGTATTTCAAGAGAAGAACAAGATACATTTGCTGTAAACTCACAACATAAAGCAGTACGTGCACAGCAA |
| **36** | **>arcC_2**  TTATTAATCCAACAAGCTAAATCGAACAGTGACACAACGCCGGCAATGCCATTGGATACTTGTGGTGCAATGTCACAAGGTATGATAGGCTATTGGTTGGAAACTGAAATCAATCGCATTTTAACTGAAATGAATAGTGATAGAACTGTAGGCACAATCGTAACACGTGTGGAAGTAGATAAAGATGATCCACGATTTGATAACCCAACTAAACCAATTGGTCCTTTTTATACGAAAGAAGAAGTTGAAGAATTACAAAAAGAACAGCCAGGCTCAGTCTTTAAAGAAGATGCAGGACGTGGTTATAGAAAAGTAGTTGCGTCACCACTACCTCAATCTATACTAGAACACCAGTTAATTCGAACTTTAGCAGACGGTAAAAATATTGTCATTGCATGCGGTGGTGGCGGTATTCCAGTTATAAAAAAAGAAAATACCTATGAAGGTGTTGAAGCG  **>aroE_2**  AATTTTAATTCTTTAGGATTAGATGATACTTATGAAGCTTTAAATATTCCAATTGAAGATTTTCATTTAATTAAAGAAATTATTTCAAAAAAAGAATTAGATGGCTTTAATATCACAATTCCTCATAAAGAGCGTATCATACCGTATTTAGATCATGTTGATGAACAAGCGATTAATGCAGGTGCAGTTAACACTGTTTTGATAAAAGATGGCAAGTGGATAGGGTATAATACAGATGGTATTGGTTATGTTAAAGGATTGCACAGCGTTTATCCAGATTTAGAAAATGCATACATTTTAATTTTGGGAGCAGGTGGTGCAAGTAAAGGTATTGCTTATGAATTAGCAAAATTTGTAAAGCCCAAATTAACTGTTGCGAATAGAACGATGGCTCGTTTTGAATCTTGGAATTTAAATATAAACCAAATTTCATTGGCAGATGCTGAAAAGTATTTA  **>glpF_2**  GGTGCTGATTGGATTGTCATCACAGCTGGATGGGGATTAGCGGTTACAATGGGTGTGTATGCTGTTGGTCAATTCTCAGGTGCACATTTAAACCCAGCGGTGTCTTTAGCTCTTGCATTAGACGGAAGTTTTGATTGGTCATTAGTTCCTGGTTATATTGTTGCTCAAATGTTAGGTGCAATTGTCGGAGCAACAATTGTATGGTTAATGTACTTGCCACATTGGAAAGCGACAGAAGAAGCTGGCGCGAAATTAGGTGTTTTCTCTACAGCACCGGCTATTAAGAATTACTTTGCCAACTTTTTAAGTGAAATTATCGGAACAATGGCATTAACTTTAGGTATTTTATTTATCGGTGTAAACAAAATTGCTGATGGTTTAAATCCTTTAATTGTCGGAGCATTAATTGTTGCAATCGGATTAAGTTTAGGCGGTGCTACTGGTTATGCAATCAACCCAGCACGT  **>gmk_2**  CGAATATTTGAAGATCCAAGTACATCATATAAGTATTCTATTTCAATGACAACACGTCAAATGCGTGAAGGTGAAGTTGATGGCGTAGATTACTTTTTTAAAACTAGGGATGCGTTTGAAGCTTTAATTAAAGATGACCAATTTATAGAATATGCTGAATATGTAGGCAACTATTATGGTACACCAGTTCAATATGTTAAAGATACAATGGACGAAGGTCATGATGTATTTTTAGAAATTGAAGTAGAAGGTGCAAAGCAAGTTAGAAAGAAATTTCCAGATGCGTTATTTATTTTCTTAGCACCTCCAAGTTTAGATCACTTGAGAGAGCGATTAGTAGGTAGAGGAACAGAATCTGATGAGAAAATACAAAGTCGTATTAACGAAGCACGTAAAGAAGTCGAAATGATGAATTTA  **>pta_3**  GCAACACAATTACAAGCAACAGATTATGTTACACCAATCGTGTTAGGTGATGAGACTAAGGTTCAATCTTTAGCGCAAAAACTTAATCTTGATATTTCTAATATTGAATTAATTAATCCTGCGACAAGTGAATTGAAAGCTGAATTAGTTCAATCATTTGTTGAACGACGTAAAGGTAAAACGACTGAAGAACAAGCACAAGAATTATTAAACAATGTGAACTACTTCGGTACAATGCTTGTTTATGCTGGTAAAGCAGATGGTTTAGTTAGTGGTGCAGCACATTCAACAGGCGACACTGTGCGTCCAGCTTTACAAATCATCAAAACGAAACCAGGTGTATCAAGAACATCAGGTATCTTCTTTATGATTAAAGGTGATGAACAGTACATCTTTGGTGATTGTGCAATCAATCCAGAACTTGATTCACAAGGACTTGCAGAAATTGCAGTAGAAAGTGCAAAATCAGCATTA  **>tpi_3**  CACGAAACAGATGAAGAAATTAACAAAAAAGCGCACGCTATTTTCAAACATGGAATGACTCCAATTATTTGTGTTGGTGAAACAGACGAAGAGCGTGAAAGTGGTAAAGCTAACGATGTTGTAGGTGAGCAAGTTAAGAAAGCTGTTGCAGGTTTATCTGAAGATCAACTTAAATCAGTTGTAATTGCTTATGAACCAATCTGGGCAATCGGAACTGGTAAATCATCAACATCTGAAGATGCGAATGAAATGTGTGCATTTGTACGTCAAACTATTGCTGACTTATCAAGCAAAGAAGTATCAGAAGCAACTCGTATTCAATATGGTGGTAGTGTTAAACCTAACAACATTAAAGAATACATGGCACAAACTGATATTGATGGGGCATTAGTAGGTGGCGCA  **>yqiL_2**  GCGTTTAAAGACGTGCCAGCCTATGATTTAGGTGCGACTTTAATAGAACATATTATTAAAGAGACGGGTTTGAATCCAAGTGAGATTAATGAAGTCATCATCGGTAACGTACTACAAGCAGGACAAGGACAAAATCCAGCACGAATTGCTGCTATGAAAGGTGGCTTGCCAGAAACAGTACCTGCATTTACAGTGAATAAAGTATGTGGTTCTGGGTTAAAGTCGATTCAATTAGCATATCAATCTATTGTGACTGGTGAAAATGACATCGTGCTAGCTGGCGGTATGGAGAATATGTCTCAATCACCAATGCTTGTCAACAACAGTCGCTTTGGTTTTAAAATGGGACATCAATCAATGGTTGATAGCATGGTATATGATGGTTTAACAGATGTATTTAATCAATATCATATGGGTATTACTGCTGAAAATTTAGTAGAGCAATATGGTATTTCAAGAGAAGAACAAGATACATTTGCTGTAAACTCACAACAAAAAGCAGTACGTGCACAGCAA |
| **1637** | **>arcC_1**  TTATTAATCCAACAAGCTAAATCGAACAGTGACACAACGCCGGCAATGCCATTGGATACTTGTGGTGCAATGTCACAGGGTATGATAGGCTATTGGTTGGAAACTGAAATCAATCGCATTTTAACTGAAATGAATAGTGATAGAACTGTAGGCACAATCGTTACACGTGTGGAAGTAGATAAAGATGATCCACGATTCAATAACCCAACCAAACCAATTGGTCCTTTTTATACGAAAGAAGAAGTTGAAGAATTACAAAAAGAACAGCCAGACTCAGTCTTTAAAGAAGATGCAGGACGTGGTTATAGAAAAGTAGTTGCGTCACCACTACCTCAATCTATACTAGAACACCAGTTAATTCGAACTTTAGCAGACGGTAAAAATATTGTCATTGCATGCGGTGGTGGCGGTATTCCAGTTATAAAAAAAGAAAATACCTATGAAGGTGTTGAAGCG  **>aroE_4**  AATTTTAATTCTTTAGGATTAGATGATACTTATGAAGCTTTAAATATTCCAATTGAAGATTTTCATTTAATTAAAGAAATTATTTCGAAAAAAGAATTAGATGGCTTTAATATCACAATTCCTCATAAAGAACGTATCATACCGTATTTAGATTATGTTGATGAACAAGCGATTAATGCAGGTGCAGTTAACACTGTTTTGATAAAAGATGGCAAGTGGATAGGGTATAATACAGATGGTATTGGTTATGTTAAAGGATTGCACAGCGTTTATCCAGATTTAGAAAATGCATACATTTTAATTTTGGGCGCAGGTGGTGCAAGTAAAGGTATTGCTTATGAATTAGCAAAATTTGTAAAGCCCAAATTAACTGTTGCGAATAGAACGATGGCTCGTTTTGAATCTTGGAATTTAAATATAAACCAAATTTCATTGGCAGATGCTGAAAAGTATTTA  **>glpF_1**  GGTGCTGATTGGATTGTCATCACAGCTGGATGGGGATTAGCGGTTACAATGGGTGTGTTTGCTGTCGGTCAATTCTCAGGTGCACATTTAAACCCAGCGGTGTCTTTAGCTCTTGCATTAGACGGAAGTTTTGATTGGTCATTAGTTCCTGGTTATATTGTTGCTCAAATGTTAGGTGCAATTGTCGGAGCAACAATTGTATGGTTAATGTACTTGCCACATTGGAAAGCGACAGAAGAAGCTGGCGCGAAATTAGGTGTTTTCTCTACAGCACCGGCTATTAAGAATTACTTTGCCAACTTTTTAAGTGAGATTATCGGAACAATGGCATTAACTTTAGGTATTTTATTTATCGGTGTAAACAAAATTGCCGATGGTTTAAATCCTTTAATTGTCGGAGCATTAATTGTTGCAATCGGATTAAGTTTAGGCGGTGCTACTGGTTATGCAATCAACCCAGCACGT  **>gmk_127**  CGAATATTTGAAGATCCAAGTACATCATATAAGTATTCTATTTCAATGACAACACGTCAAATGCGTGAAGGTGAAGTTGATGGCGTAGATTACTTTTTTAAAACTAGGGATGCGTTTGAAGCTTTAATTAAAGATGACCAATTTATAGAATATGCTGAATATGTAGGCAACTATTATGGTACACCAGTTCAATATGTTAAAGATACAATGGACGAAGGTCATGATGTATTTTTAGAAATTGAAGTAGAAGGTGCAAAGCAAGTTAGAAAGAAATTTCCAGATGCGTTATTTATTTTCTTAGCACCTCCAAGTTTAGATCACCTGAGAGAGCGATTAGTAGGTAGAGGAACAGAATCCAATGAGAAAATACAAAGTCGTATTAACGAAGCGCGTAAAGAAGTTGAAATGATGAATTTA  **>pta_12**  GCAACACAATTACAAGCAACAGATTATGTTACACCAATCGTGTTAGGTGATGAGACTAAGGTTCAATCTTTAGCGCAAAAACTTGATCTTGATATTTCTAATATTGAATTAATTAATCCTGCGACAAGTGAATTGAAAGCTGAATTAGTTCAATCATTTGTTGAACGACGTAAAGGTAAAGCGACTGAAGAACAAGCACAAGAATTATTAAACAATGTGAACTACTTCGGTACAATGCTTGTTTATGCTGGTAAAGCAGATGGTTTAGTTAGTGGTGCAGCACATTCAACAGGAGACACTGTGCGTCCAGCTTTACAAATCATCAAAACGAAACCAGGTGTATCAAGAACATCAGGTATCTTCTTTATGATTAAAGGTGATGAACAATACATCTTTGGTGATTGTGCAATCAATCCAGAACTTGATTCACAAGGACTTGCAGAAATTGCAGTAGAAAGTGCAAAATCAGCATTA  **>tpi_1**  CACGAAACAGATGAAGAAATTAACAAAAAAGCGCACGCTATTTTCAAACATGGAATGACTCCAATTATTTGTGTTGGTGAAACAGACGAAGAGCGTGAAAGTGGTAAAGCTAACGATGTTGTAGGTGAGCAAGTTAAGAAAGCTGTTGCAGGTTTATCTGAAGATCAACTTAAATCAGTTGTAATTGCTTATGAGCCAATCTGGGCAATCGGAACTGGTAAATCATCAACATCTGAAGATGCAAATGAAATGTGTGCATTTGTACGTCAAACTATTGCTGACTTATCAAGCAAAGAAGTATCAGAAGCAACTCGTATTCAATATGGTGGTAGTGTTAAACCTAACAACATTAAAGAATACATGGCACAAACTGATATTGATGGGGCATTAGTAGGTGGCGCA  **>yqiL_10**  GCGTTTAAAGACGTGCCAGCCTATGATTTAGGTGCGACTTTAATAGAACATATTATTAAAGAGACGGGTTTGAATCCAAGTGAGATTGATGAAGTTATCATCGGTAACGTACTACAAGCAGGACAAGGACAAAATCCAGCACGAATTGCTGCTATGAAAGGTGGCTTGCCAGAAACAGTACCTGCATTTACAGTGAATAAAGTATGTGGTTCTGGGTTAAAGTCGATTCAATTAGCATATCAATCTATTGTGACTGGTGAAAATGACATCGTGCTAGCTGGCGGTATGGAGAATATGTCTCAGTCACCAATGCTTGTCAACAACAGTCGCTTCGGTTTTAAAATGGGACATCAATCAATGGTTGATAGCATGGTATATGATGGTTTAACAGATGTATTTAATCAATATCATATGGGTATTACTGCTGAAAATTTAGTAGAGCAATATGGTATTTCAAGAGAAGAACAAGATACATTTGCTGTAAACTCACAACATAAAGCAGTACGTGCACAGCAA |
